# Ultrasensitive Detection of Biomarkers in a Color‐Switchable Microcavity‐Reactor Laser

**DOI:** 10.1002/advs.202202326

**Published:** 2022-06-08

**Authors:** Ran Li, Zongpeng Song, Haiou Zhu, Fanglin Zhang, Lingling Chen, Cun‐Zheng Ning, Shuangchen Ruan

**Affiliations:** ^1^ College of New Materials and New Energies Shenzhen Technology University Shenzhen 518118 China; ^2^ School of Chemistry Chemical Engineering and Life Sciences Wuhan University of Technology Wuhan 430070 China; ^3^ College of Health and Environmental Engineering Shenzhen Technology University, China Shenzhen 518118 China; ^4^ Department of Electronic Engineering Tsinghua University Beijing 100084 China

**Keywords:** biomarkers, color‐switchable microlasers, ultrasensitive detection, visual distinguishability

## Abstract

Early detection and diagnosis are vitally important in reducing the mortality rate of fatal diseases but require highly sensitive detection of biomarkers. Presently, detection methods with the highest sensitivity require in vitro processing, while in vivo compatible fluorescence detections require a much higher concentration of biomarkers or limit of detection (LOD). In this paper, a fundamentally new strategy for ultrasensitive detection based on color‐switchable lasing with a cavity‐enhanced reduction of LOD is demonstrated, down to 1.4 × 10^−16^ mg ml^−1^ for a quantitative detection, lower than both the fluorescence method and plasmonic enhanced method. For a qualitative or a yes/no type of detection, the LOD is as low as 10^–17^ mg ml^−1^. The approach in this work is based on a dye‐embedded, in vivo compatible, polystyrene‐sphere cavity, penetrable by biomarkers. A polystyrene sphere serves the dual roles of a laser cavity and an in vivo bio‐reactor, in which dye molecules react with a biomarker, reporting biomarker information through lasing signals. The cavity‐enhanced emission and lasing with only a single biomarker molecule per cavity allow improved visual distinguishability via color changes. Furthermore, when combined with a narrow‐band filter, the color‐switchable lasers act as an “on‐off” logic signal and can be integrated into multiplexing detection assay biochips.

## Graphical abstract

A permeable color‐switchable whispering‐gallery‐mode (WGM) microlaser for ultrasensitive detection of biomarker is developed as illustrated in the scheme above. A self‐assembled, dye‐embedded microsphere cavity is permeated by target biomarkers, then the dye‐biomarker chemical bonding results in a large wavelength shift of lasing wavelength. Importantly, the more sensitive concentration dependence allows even visual distinguishability of small changes in biomarker concentration, such that “normal” status or even different stages of the disease (“abnormal” status) can be visually discriminated. Due to the narrow cavity modes and largely shifted spectral peaks, the microlaser output from the microsphere can act as “normal–abnormal” logic signal to integrate into multiplexing detection assays.

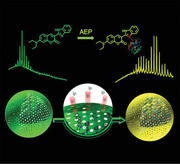

## 1 Introduction

Biomarkers in tissues, serum, or body fluids contain information from cell in the human body, providing critical reporters/indicators for different stages of diseases.^[^ ^1^, ^2^ ^]^ For early‐stage detection, dramatic improvement in detection sensitivity is necessary to detect biomarkers at ultralow levels. Take Alzheimer's disease (AD) as an example, it develops over a long preclinical period for several decades before mild cognitive impairment occurs.^[^ ^4^ ^]^ The earliest evidence of AD pathophysiological changes lies below the current detection threshold of biomarkers, or LOD. Reducing LOD may result in a much earlier treatment by years both for AD and cancers^[^ ^3^, ^4^ ^]^ and can potentially save many more lives. A great deal of effort has been devoted to the exploration of new technologies to reduce the LOD as summarized in Supporting Information Table S1, such as optical,^[^ ^1^, ^5^, ^6^, ^7^, ^8^, ^9^, ^10^, ^11^, ^12^ ^]^ mechanical,^[^ ^13^, ^14^, ^15^, ^16^, ^17^ ^]^ electrical,^[^ ^2^, ^18^ ^]^ and other detection methods.^[^ ^19^ ^]^ So far, the most promising approach is plasmonic enzyme‐linked immunosorbent assay (ELISA) that achieved the lowest LOD of 10^–15^ mg ml^−1^ through the change of absorption spectra^[^ ^12^ ^]^ but is incompatible with in vivo detection. For the conventional optical methods that are in vivo compatible, the further improvement on LOD is limited by the broad bandwidth of absorption or emission spectrum. The fluorescence method is capable of differentiating molecules with similar structures with a LOD of ≈2 × 10^–7^ mg ml^−1^ for homocysteine^[^ ^20^ ^]^ and is far from being sufficient to meet the demands for early detection.^[^ ^21^ ^]^

To reduce LOD, weak signals from biomarkers of low concentration should be preferably exponentially amplified. Biomarkers such as nucleic acids may exponentially replicate themselves to increase their concentration,^[^ ^22^, ^23^ ^]^ while other biomarkers such as proteins cannot be “amplified” in this way. In such cases, optical amplification is an appealing alternative. Currently, optical methods are limited by the broadband features of absorption and emission. Optical amplification in a resonant cavity can lead to significant spectral narrowing through the Purcell enhancement or lasing, potentially leading to a significant reduction of LOD. Various micro/nanocavity‐based detection schemes have been reviewed,^[^ ^24^, ^25^, ^26^ ^]^ and typical micro/nanocavity‐based detection is listed in Table S2 Supporting Information^.[^ ^27^, ^28^, ^29^, ^30^ ^]^ Si‐microtoroid resonator biological sensor for interleukin‐2 was successfully developed with a resonant wavelength shift ≤1 pm.^[^ ^28^ ^]^ And Si‐microtoroid substrates were developed for biomolecular recognition with a frequency splitting of tens of MHz^[^ ^29^ ^]^ with a LOD of ≈7.5 × 10^–14^ mg ml^−1^ (10^–17^ mol L^−1^). Nanocavity was used to detect explosives of 2,4‐dinitrotoluene and 2,4,6‐trinitrotoluene through changes in laser intensity without a wavelength shift.^[^ ^27^, ^30^ ^]^ As the detection‐induced spectral changes in these methods are minimal, expensive equipment with extremely high spectral precision is indispensable, and no real‐time qualitative readout could be realized.

**Figure 1:**
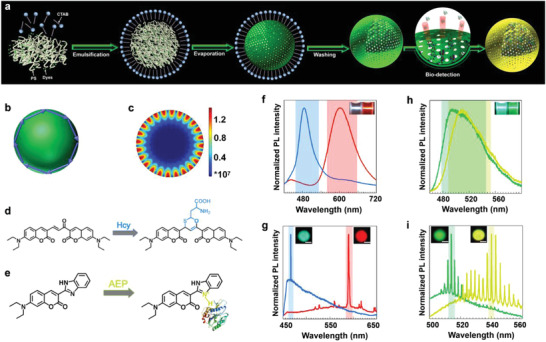
Preparation of polystyrene sphere (PSS) samples and detection mechanism. a) Schematic of the formation of microspheres made of polystyrenes mixed with dye, and the permeation and detection of biomarker molecules. b) Schematic of the light paths and mode formation in a sphere. c) The electric field intensity distribution of a WGM in a microcavity simulated by COMSOL; Reaction mechanism of DC with Hcy d) and C7 with AEP e). Photoluminescence (PL) spectra of DC with (blue) and without (red) Hcy f) and DC+PSS with (blue) and without (red) Hcy g). PL spectra of C7 with (green) and without (cyan) AEP h) and C7+PSS with (green‐yellow) and without (green) AEP i) excited by a focused pulsed laser beam (400 nm). Photographs of DC before and after the addition of Hcy (f inset) and C7 before and after the addition of AEP (h inset) excited by a focused pulsed laser beam (400 nm); confocal microscopy images of DC+PSS sphere before and after the addition of Hcy (g inset) and C7+PSS sphere before and after the addition of AEP (i inset) excited by a mercury lamp. The scale bar of this confocal microscope image is 5 µm.

In this paper, a novel approach is introduced to reduce the LOD significantly beyond the current limit through exponential amplification of emission within a bio‐compatible and permeable WGM cavity (**Figure** **1**). The approach is based on microsphere cavities formed by bio‐compatible polystyrene (PS) mixed with dyes, penetrated by biomarker molecules when submerged in the biomolecular solution. When biomarkers interact with the chemo‐responsive dyes (Figure 1d,e), intramolecular charge‐transfer modifies the electronic structure of dyes, resulting in the shift of emission wavelengths (Figure 1f,h). This is the basic principle of conventional fluorescence‐based detection. But such broadband emission results in weak signal and poorly separated shift as shown in Figure 1h. Inside a cavity, the broad spectrum of emission is compressed into narrow WGM modes (Figure 1g,i) with strong Purcell enhancement. At high optical pumping levels, such WGM modes start to lase, leading to further amplification of fluorescence signals. Due to the dramatic cavity enhancement, the strong photoluminescence (PL) signal results in the sensitive detection of much lower concentrations. The narrow and widely separated spectral lines of cavity modes (Figure 1g,i) allow much simpler and cheaper detection with a simple filter without the need for a spectrometer. Importantly, the enhanced intensity and more sensitive concentration dependence allow even visual distinguishability (Figure 3f,l) of small changes in biomarker concentration, such that health status or even different stages of the disease can be visually discriminated.

To demonstrate the approach, we use as biomarker examples homocysteine (Hcy), cysteine (Cys) and glutathione (GSH), and protease protein asparaginyl endopeptidase (AEP). The biological thiols (Hcy, Cys, and GSH) have similar structures and play important roles in various physiological processes.^[^ ^7^, ^31^ ^]^ Abnormal levels of these biological thiols are indicative of diseases such as liver damage, cancer, and osteoporosis. AEP is highly activated and overexpressed in human brains with AD and Parkinson's disease.^[^ ^32^, ^33^ ^]^ The concentration of biomarkers is beyond the upper limit and would cause the disease, defined as abnormal biomarkers. The results demonstrate a decrease of LOD by 5 (Hcy) or ≈6 (activated AEP) orders of magnitude using the microsphere cavity‐based detection compared to the conventional PL approach without a cavity. The LODs for Hcy and activated AEP are 10^–9^ and 1.4 × 10^−16^ mg ml^−1^, respectively. The lowest LOD for activated AEP is lower than that of the current best approach, plasmonic ELISA (10^–15^ mg ml^−1^).

## 2 Results and Discussion

### 2.1 Detection Mechanism

Structurally, fluorescent sensors here contain a probe moiety to provide molecular recognition through interactions with special analytes and a reporter moiety for optical transduction through fluorophores.^[^ ^34^ ^]^ For coumarin derivatives (Figure S1 Supporting Information), electron‐donor (D) groups in position 7 and electron‐acceptor (A) groups in position 3 bring bathochromic shift emission.^[^ ^35^ ^]^ When reacting with biomarkers, the electronegativity of D or A groups is then modulated, tuning the emission wavelength of the coumarin derivatives. Accordingly, 7‐diethylaminocoumarin‐3‐carbaldehyde‐3‐acetyl‐7‐(diethylamino) coumarin (DC, Supporting Information Figures S3, and S4) and 3‐(2‐benzimidazolyl)‐7‐(diethylamino) coumarin (C7, Supporting Information, Figures S5, and S6) were selected as the model compounds.

DC was synthesized and characterized as shown in Figure S2, Supporting Information. DC molecules show a strong emission peak centered at ≈600 nm and a weak emission peak at ≈450 nm (Figure 1f). When reacting with Hcy, a stable six‐membered ring structure is formed between the conjugated ketene moiety of DC and the mercaptoethyl group of Hcy (Figure 1d). The conjugated structure of DC is thus interrupted and the strong emission peak at ≈600 nm (red) is quenched, enhancing the emission peak at ≈480 nm (Figure 1f). While for Cys (with only one less methylene than Hcy), when reacting with DC, no obvious emission shift was detected because of the instability of five‐membered rings between the conjugated ketene moiety of DC and the mercaptoethyl group of Cys.^[^ ^36^ ^]^ Due to the large steric hindrance of GSH, the kinetic process of the Michael addition between DC and GSH is greatly weakened, so Hcy could be selectively detected from Cys and GSH by DC probe (Supporting Information, Figures S7, and S8). AEP macromolecule was activated in acid fluid and the protonic acid group in the activated AEP could interact with the lone‐pair electrons on the nitrogen atom in the benzimidazole group of C7 (Supporting Information, Figures S9, and S10). The interaction between C7 and the protonic acid group in the activated AEP can reduce the HOMO‐LUMO gap from 3.52 to 3.32 eV (Figure S11 Supporting Information), which demonstrates the theoretical feasibility of detecting activated AEP with C7. With the addition of activated AEP (10^–6^ mg ml^−1^), the initial emission peak of C7 at ≈495 nm (cyan) changed to its protonated state at ≈515 nm (green) as shown in the inset of Figure 1h. Thus, it is believed that chemo‐responsive dyes of DC and C7 can be suitable optical probes for biomarker detection.

Different from previously reported microcavity‐ or microring‐based detection methods,^[^ ^28^, ^29^, ^37^, ^38^, ^39^, ^40^, ^41^ ^]^ the resonant cavity in this study is formed by bio‐compatible permeable polystyrene spheres (PSSs) with dyes inside the cavity^[^ ^42^, ^43^ ^]^ as shown in Figure 1a and Figure S12, Supporting Information. Dye molecules (C7 or DC) were pre‐mixed with PS chains and then the dyes are automatically embedded into the subsequently formed cavities.^[^ ^44^ ^]^ When PS chains twine together, they form PSSs with about 10 µm in diameter, with some nanoholes formed throughout the microspheres.^[^ ^45^, ^46^ ^]^ Such PSSs serve naturally as spherical microcavities with a refractive index of about 1.6^[^ ^47^ ^]^ and support WGM modes through total internal reflection as shown in Figure 1b,c and Figures S13 and S14, Supporting Information. The nanoholes or nanochannels throughout the PSS cavities allow easy permeation of small molecules or proteins into the PSS cavities to react with chemo‐responsive dyes inside the cavities. Such a PSS forms a natural bio‐reactor inside the optical cavity, and this cavity allows ultrasensitive bio‐analysis or detection based on Purcell enhancement of emission and lasing.

### 2.2 Color‐Switchable Microlasing

**Figure 2:**
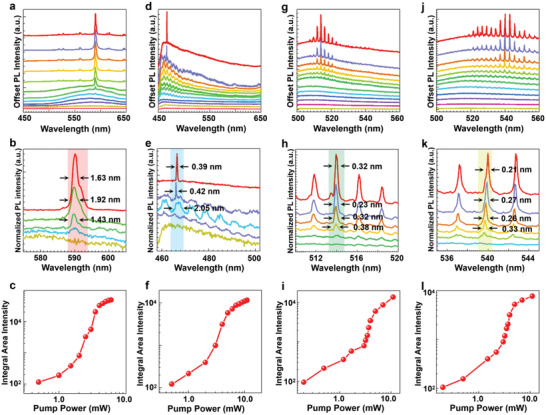
Color‐switchable microlasing. Lasing characteristics including spectral evolution with increasing pump power (a,d,g,j), the enlarged spectrum around lasing peak (b,e,h,k with pumping power as labeled and linewidth indicated in nm) and double logarithm plot of the integrated PL intensity around the strongest peak (lightly shaded area in the second row) with pump power (c,f,i,l) for the following four situations: DC+PSS before a–c) and after d–f) the addition of Hcy (10^–3^ mg ml^−1^). C7+PSS before g–i) and after j–l) the addition of AEP (10^–6^ mg ml^−1^). Pump power levels (from bottom to top): a: 0.5, 1.0, 1.5, 2.0, 2.5, 3.0, 3.5, 4.0, 4.5, 5.0, 5.5, and 6.0 mW; b: 1.0, 2.5, 3.0, 3.5, and 6.0 mW; d: 0.5, 1.0, 2.0, 3.0, 4.0, 5.0, 6.0, 7.0, 8.0, 9.0, 10.0, and 11.0 mW; e: 1.0, 2.0, 4.0, 10.0, and 11.0 mW; g: 0.2, 0.5, 1.2, 1.7, 3.0, 3.2, 3.5, 3.7, 4.0, 5.0, 7.0, and 11.0 mW; h: 3.0, 3.7, 4.0, 5.0, 7.0, and 11.0 mW; j: 0.2, 0.5, 1.2, 2.2, 3.0, 3.2, 3.5, 3.7, 4.0, 5.0, 7.0, and 11.0 mW; k: 3.2, 3.5, 4.0, 5.0, 7.0, and 11.0 mW.

The confocal microscopy images for DC embedded PSS (DC+PSS) and Hcy+DC+PSS, as well as C7+PSS and AEP+ C7+PSS are shown in the insets of Figure 1g,i, respectively, under the excitation of a mercury lamp, which shows visually distinguishable color switch with the addition of biomarkers. PL spectra were measured with the pump from a focused pulsed laser beam (400 nm) using a homemade micro‐PL system. Basic lasing features without (DC+PSS (**Figure** **2**a–c) and C7+PSS (Figure 2g–i)) and with (Hcy+DC+PSS (Figure 2d–f) and AEP+C7+PSS (Figure 2j–l)) biomarkers are shown in Figure 2. The appearance of cavity modes and spectral evolution with peak narrowing (see the first and second rows) show clear lasing behavior, especially the nonlinear increase of modal intensity, as evidenced by the typical “S”‐type behavior^[^ ^48^ ^]^ of the third row in Figure 2. The typical spectral linewidth narrowing is not always evident due to the limit of spectral resolution (larger than 0.2105 nm (grating groove 300) and 0.1028 nm (grating groove 600), respectively). The detailed spectra of peak narrowing for the strongest emission peak, as well as the first and second‐order derivatives, are shown in the Supporting Information as a further feature of the laser threshold^[^ ^48^ ^]^ for DC+PSS (Figures S16, and S17, and Table S3, Supporting Information), Hcy+DC+PSS (Figures S18, and S19 and Table S4, Supporting Information), C7+PSS (Figures S20, and S21 and Table S5, Supporting Information) and AEP+C7+PSS (Figures S22, and S23 and Table S6, Supporting Information). These spectra indicate a lasing threshold of about 3.5 mW with the lasing peak centered at ≈590 nm for DC+PSS. The addition of Hcy switches the emission peak from ≈590 nm (red) to ≈466 nm (blue) with a lasing threshold of 3 mW. A lasing threshold of about 3.5 mW with the lasing peak centered at ≈514 nm is present for C7+PSS. The addition of activated AEP switches the lasing peak from ≈514 nm (green) to ≈540 nm (green‐yellow), with a lasing threshold of 3 mW. These results clearly demonstrate the practical feasibility of color‐switchable microlasing from red to blue (Figure 1g) and green to green‐yellow (Figure 1i) as a visually distinguishable method for biomarkers detection.

### 2.3 Detection Sensitivity for Biomarkers

**Figure 3:**
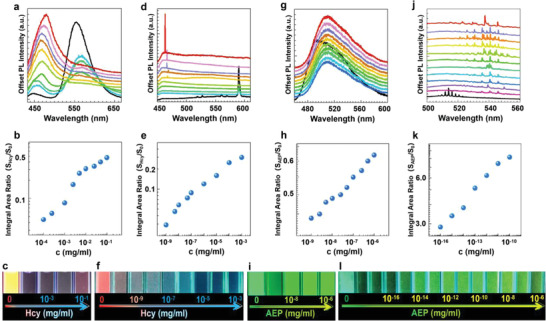
Detection sensitivity for biomarkers. Biomarker concentration‐dependent PL spectra (1st row), double‐logarithmic plot of fitted PL intensity area versus biomarker concentration (2nd row), pumped by a 400 nm femtosecond laser at 11 mW with 5 s for DC samples and 5 mW with 5 s for C7 samples. *S*_Hcy_, *S*_AEP,_ and *S*_0_ refer to the integrated intensity with and without biomarker molecules. To minimize the errors induced by the wavelength variations, integrated intensity in the wavelength around the strongest emission peak (b,e,h) and in the wavelength range of 530–550 nm (k) was used to obtain the integral area ratio. Photographs for biomarker detection with different concentration levels under the illumination of an ultraviolet lamp of 365 nm (3rd row) for the following four cases: a–c) Hcy+DC; d–f) Hcy+DC+PSS; g–i) AEP+C7; j–l) AEP+C7+PSS. Concentration levels are as follows (from bottom to top): a: 0, 10^–4^, 5 × 10^–4^, 10^–3^, 2.5 × 10^–3^, 5 × 10^–3^, 10^–2^, 2.5 × 10^–2^, 5 × 10^–2^, and 10^–1^ mg ml^−1^; d: 0, 10^–9^, 2.5 × 10^–9^, 5 × 10^–9^, 10^–8^, 2.5 × 10^–8^, 5 × 10^–8^, 10^–7^, 2.5 × 10^–7^, 5 × 10^–7^, and 10^–6^ mg ml^−1^; g: 0, 10^–9^, 5 × 10^–9^, 10^–8^, 5 × 10^–8^, 10^–7^, 10^–6^, 10^–5^, 10^–4^, and 10^–3^ mg ml^−1^; j: 0, 10^–16^, 10^–15^, 10^–14^, 10^–13^, 10^–12^, 10^–11^, 10^–10^, 10^–9^, 10^–8^, 10^–7^, and 10^–6^ mg ml^−1^.

To study the sensitivity difference between conventional fluorescent methods and the color‐switchable microlasing method, we investigated PL emission and lasing at varying levels of biomarker concentration, as shown in **Figure** **3**. From these results, it can be seen that DC and C7 can act as optical probes for the detection of Hcy and AEP with the LOD of 10^–4^ and 10^–9^ mg ml^−1^, respectively. These LODs based on conventional fluorescent detection are reduced by several orders of magnitude, if these reactions occur in the PSSs, or down to 10^–9^ and 1.4 × 10^−16^ mg ml^−1^, respectively. Based on the estimate (see Supporting Information, S8,), the lowest biomarker concentration of 10^–16^ mg ml^−1^ for AEP is equivalent to about 2 biological macromolecules per milliliter. For a PSS of 10 µm in diameter with a volume of 520 µm^–3^, the average number of PSS is roughly 9.1 × 10^3^ count ml^−1^ (see Supporting Information, S8), assuming all the AEP macromolecules eventually end up with being inside of the PSSs. This means that for AEP density below ≈4 × 10^–13^ mg ml^−1^, the number of AEP macromolecules is smaller than the number of PSS cavities. Or there is less than one AEP macromolecule per cavity on average. At the low AEP‐density limit (≈10^–16^ mg ml^‐1^), there are roughly 9100 PSS cavities trying to catch two AEP macromolecules. Therefore, the detection of a single biological macromolecule is realized at this level. In a previous report,^[^ ^32^ ^]^ Sodium Dodecyl Sulfate‐Polyacrylamide Gel Electrophoresis (SDS‐PAGE) analysis revealed that the molecular weight of one AEP_pH3.5_ was ≈36 kDa, including 2088 proteins. When an AEP macromolecule enters a PSS cavity, the dye molecules inside the cavity have enough time to react with thousands of proteins of this single AEP macromolecule, thereby changing emission wavelengths of those dye molecules that are bound to the proteins. To sum up, we have realized a remarkable single‐biomarker detection using the color‐switchable microlasing method. The LOD achieved with AEP is lower than the best of the current state‐of‐the‐art technique of ELISA.

The third row of Figure 3 shows photographs of the solution of dyes after reacting with biomarkers of varying concentration with (Figure 3f,l) and without (Figure 3c,i) PSSs. It can be observed that the presence of abnormal biomarkers is visible with and without PSSs. But the visual distinguishability extends to a much smaller concentration from 10^–4^ (Figure 3c) to 10^–10 ^mg ml^−1^ (Figure 3f) for Hcy and from 10^–9^ (Figure 3i) to 10^–16^ mg ml^−1^ for AEP (Figure 3l) with PSSs through the cavity enhancement. Such visually distinguishable color changes through a change of one order of magnitude of the biomarkers represent one of the most precise detection methods through simple visual inspection by the naked eye. Qualitative detection of biomarkers can be realized with a naked eye under the irradiation of a simple UV LED as shown in Figure 3c,f,i,l. We note that the color distinguishability with the naked eyes is actually better than that from the photographs due to the limitation of the color scheme in the current photograph technology. Such low cost for qualitative detection of biomarker could fundamentally impact early‐stage disease detection and diagnostics.

### 2.4 Statistical Analysis of Biomarker AEP

**Figure 4:**
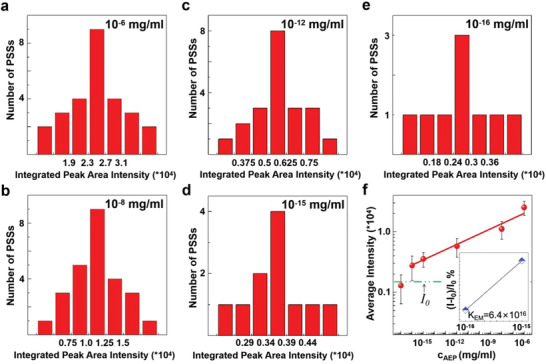
Statistical analysis and quantitative detection of biomarker AEP. a–e) histograms at five different concentration levels of AEP. The number of PSSs measured are *n*_1_ = 27, *n*_2_ = 26, *n*_3_ = 21, *n*_4_ = 11, and *n*_5_ = 9 for concentration level at 10^–6^, 10^–8^, 10^–12^, 10^–15^, and 10^–16^ mg ml^−1^, respectively. At each concentration level, the total emission intensity from cavity modes from 530 to 550 nm are integrated and numbers of intensity counts are used to obtain histograms a‐e. f) average intensity versus AEP concentration. The calculation of error bar of each concentration is shown in Table S11, Supporting Information. The green line represents the lasing intensity without biomarkers (*I_0_*). The average intensity at 10^−17^ mg ml^−1^ is below *I*_0_ and is not considered for quantitative measurement. The inset shows the enhancement ratio (see Supporting Information S10) of PL intensity (monitored at 530–550 nm) of AEP+C7+PSS as a function of AEP concentration.

To determine quantitatively the concentration of biomarkers with spectral information, and to eventually relate such concentration level to the severity of a disease, it is shown in **Figure** **4** results of statistical analysis are based on the spectral measurements (Supporting Information, Figure S28–S34) of large numbers of PSS cavities at a series of concentration levels. Such statistical analysis is necessary because of the unavoidable size non‐uniformity of PSSs and the variation in the coupling between biomarkers and PSS cavities. Figure 4a–e shows the histograms of these measurements (see captions to Figure 4 for details). We extracted the average intensity from these histograms and plotted in Figure 4f versus AEP concentration. As shown in Figure 4f, the average intensity depends on the AEP concentration (*c*_AEP_). The data in Figure 4 and the associated scaling relation serve as the basis for a quantitative detection of AEP down to 10^–16^ mg ml^−1^. The last data point at the AEP density of 10^−17^ mg ml^−1^ has too large an error due to the smaller number of experiments. To accurately express the detection sensitivity of biomarkers, the LOD was calculated using the 3*σ* equation,^[^ ^49^ ^]^ and a LOD of 1.4 × 10^−16^ mg ml^−1^ was calculated (Supporting Information, S10). In addition, the spectral measurements at the AEP level of 10^–17^ mg ml^−1^ were also performed and shown (Figure S33, Supporting Information). We see clearly there that the presence of this low level of AEP macromolecules still produces relatively strong spectral peaks in the long‐wavelength range (say, 530–550 nm) compared to short‐wavelength range (say, 510–520 nm) whereas relatively weak peaks exist without AEP macromolecules. Therefore, even though the error is large at such low AEP levels for quantitative measurement, the presence of relatively strong mode peaks in the long‐wavelength range may serve as a yes/no‐type of detection of AEP macromolecules.

## 3 Conclusions

To summarize, we have demonstrated a fundamentally new method of ultrasensitive detection of biomarkers using bio‐compatible PS microspheres. The approach overcomes one of the key drawbacks of conventional fluorescence‐based detection. PL spectra in the fluorescence method often have a large spectral overlap before and after reaction with biomarkers (see, especially the overlap of green and cyan spacing in Figure 1h). For the color‐switchable microlasing approach, there is no spectral overlap before and after the addition of biomarker (Figure 1g,i). This makes the detection of biomarkers much simpler, less time‐consuming, and less expensive for a qualitative detection, than other high‐sensitivity detections, especially in comparison with other micro‐cavity‐based approaches where tiny spectrometer shift is involved and extremely precise and expensive spectrometers are required.

Importantly, the approach integrates the dual functionalities of PS microcavities in the most natural manner: As a reactor for biomarkers to react with dye molecules and as a micro‐resonator for enhanced emission and lasing that reports the biomarker concentration. Several important features of this approach and potential future perspectives are worth noting: 1) The chemical binding of biomarkers with dye molecules for a biosensor in our case leads to large shifts of lasing wavelength on the order of 20–30 nm (C7+PSS) or 150 nm (DC+PSS); The large shifts require only an inexpensive spectrometer or a simple band filter to resolve, even allows determination by the naked eye. 2) Beyond ultrasensitive detection through quantitative measurement of lasing intensity (Figure 4f) down to 10^−16^ mg ml^−1^, the strongly enhanced emission and lasing also allow easily recognizable yes‐no detection of biomarkers (visually qualitative detection) down to 10^−17^ mg ml^−1^ level through the presence of relatively strong spectral peaks or by a naked‐eye inspection of color change. 3) Due to the narrow cavity modes and widely separated spectral peaks, the microlaser output from the PSSs can act as “on–off” (“normal–abnormal”) logic signal to integrate into multiplexing detection assays or a bio‐chip for detection of various biomarkers with massive functional groups for intelligent analysis of disease screening. 4) The simplicity of submerging into a testing environment, letting the penetration of biomarkers into the PS cavities, and then reacting inside the PS cavities is a universal approach that can be used for ultrasensitive detection of many other biomarkers. 5) Dyes have been widely used in in vivo fluorescence imaging, the microlaser‐based approach could be performed for in‐vivo detection for biomarkers, and more importantly, provide a new imaging avenue with much higher intensity and larger contrast. All these scenarios offer exciting future opportunities for biomedical detection and early diagnostics.

## 4 Experimental Section

### Test Conditions of C7 for the Detection of AEP (Without PSSs)

C7 (density: 10^–2^ mg ml^−1^, and total quantity: 1 ml) was added into AEP solution of different concentrations (10^–6^‐10^–9^ mg ml^−1^) at room temperature for several minutes. The corresponding fluorescence spectra of C7 with AEP were measured in a Zolix optical system with a picosecond laser (375 nm) and a homemade micro‐PL system (Figure S15, Supporting Information), respectively.

### Test Conditions of DC for the Detection of Hcy (Without PSSs)

Hcy was probed via fluorescence spectroscopy in DMSO‐PBS (pH 7.4, 10 mM, v/v, 5/5) solution at room temperature. DC (density: 10^–2^ mg ml^−1^, and total quantity: 1 ml) was added into Hcy solution of different concentrations (10^–1^‐10^–4^ mg ml^−1^) at room temperature for dozens of minutes. The corresponding fluorescence spectra of DC with Hcy were measured in a Zolix optical system with a picosecond laser (375 nm) and a homemade PL system, respectively.

### Test Conditions of C7+PSS for the Detection of Activated AEP

The optically pumped lasing measurements for the samples were conducted on a homemade micro‐PL system. The excitation pulses (400 nm) were generated from an optical parametric amplifier pumped by a regenerative amplifier (Legend Elite‐1K‐HE; 800 nm, 35 fs, 3 mJ), which was in turn seeded by a mode‐locked Ti: sapphire laser. The excitation laser was filtered with a 435 nm long‐pass filter and then focused down to a 6 µm diameter spot through an objective lens (Nikon CFLU Plan, ×20, N.A. = 0.5) as a nearly uniform pump source. The measurement was done under the same conditions including the focus of the objective lens, and alignment of the optical path. The measurement process is as follows: C7 + PSS (1 ml) was added into activated AEP solution of different concentrations (10^–6^–10^–17^ mg ml^−1^, 1 ml) at room temperature, then vigorously shaken for several minutes and standing for about 20 min. The solution was then dropped onto a glass slide and transferred to the homemade micro‐PL system. For the measurements of lasing features, a color‐switchable AEP + C7 + PSS was selected each time and pumped by the 400 nm fs laser at an average power of 5 mW.

### Test Conditions of DC+PSS for the Detection of Hcy

The system of optically pumped lasing measurements was the same as described above. DC+PSS (1 ml) was added into Hcy solution of different concentrations (10^–3^–10^–9^ mg ml^−1^, 1 ml) at room temperature for dozens of minutes.

## Conflict of Interest

The authors declare no conflict of interest.

## Author Contributions

R.L. and Z.P.S. contributed equally to this work. S.C.R., C.Z.N. and H.O.Z. supervised the overall project. R.L. prepared the PSS spheres and performed the detection experiment, Z.P.S. built the homemade PL measurement system. F.L.Z. prepared the DC molecules. L.L.C. measured the confocal microscopy images. R.L., H.O.Z., and C.Z.N. analyzed the data and wrote the manuscript.

## Supporting information

Supporting Information

## Data Availability

The data that support the findings of this study are available from the corresponding author upon reasonable request.
