# Supplementary material for: Ultrasensitive Detection of Biomarkers in a Color‐Switchable Microcavity‐Reactor Laser
Source: Adv Sci (Weinh). 2022 Jun 8;9(23):2202326. doi: 10.1002/advs.202202326 (PMC9376852; doi:10.1002/advs.202202326)
Supplement: Supplementary file 1 — Supporting Information [file ADVS-9-2202326-s001.pdf]

Supporting Information

Ultrasensitive Detection of Biomarkers in a Color-Switchable Microcavity-Reactor Laser

Ran Li,# Zongpeng Song,# Haiou Zhu,* Fanglin Zhang, Lingling Chen, Cun-Zheng Ning,* and Shuangchen Ruan*

Contents

I. Materials and Methods.

Materials

Characterization

Calculation Methods

II. Figures and Tables.

S1. Summary of various technologies/methods for detection.

S2. Introduction of coumarin derivatives (DC and C7).

S3. Introduction of biomarkers (Hcy and AEP).

S4. C7 Calculation.

S5. Fabrication processes of PSSs and some details.

S6. Lasing characteristics and some details of dye+PSS.

S7. Details of PL spectra with detection sensitivity.

S8. Calculation of number (N) of PSS and biomarkers.

S9. Lasing spectra of biomarker AEP of different concentrations.

S10. LOD calculation.

I. Materials and Methods

Materials: The chemical materials of polystyrene (PS, M.W. 250,000; CAS number, 9003-53-6), coumarin 7 (C7, CAS number, 27425-55-4), homocysteine (Hcy, CAS number, 454-29-5) were purchased from Acros Organics. The dye material of DC was synthesized according to the previous reports[1] (details in Figure S7 below). The chemical solution of dichloromethane (CH_2Cl_2, CAS number, 75-09-2), dimethyl sulfoxide (DMSO, CAS number, 67-68-5) and N,N-dimethylformamide (DMF, CAS number, 68-12-2), and the physiological

solution of phosphate buffer solution (PBS, 1×) were purchased from Shanghai Macklin Biochemical Technology Co., Ltd. The biological agent of AEP was purchased from Biotech. Com. All these chemicals were used without further treatment.

Characterization: The morphology of the composite microspheres was examined through scanning electron microscopy. The absorption spectra were measured on a UV-visible spectrometer (UV 2600). The fluorescence spectra were measured on a fluorescence spectrometer with a Zhuo Lihan optical system. Bright-field optical images and fluorescence microscopy images were acquired using inverted fluorescence microscope under the excitation of a mercury lamp (330-380 nm).

In the pump-probe set up, a pump pulse excites electrons from the valence band into the conduction band of samples. We recorded the different transmission date with a white light probe beam, the change in absorbance difference (ΔA) (difference between absorption with/without of the pump light) is recorded to understand the carrier dynamics process, where are the intensity of the probe pulse after the sample with pump off and pump on, respectively.

Calculation Methods: Theoretical calculations were carried out with the Gaussian 09 software. The geometry optimizations were performed by the B3LYP function with 6-311(d, p) basis sets. After the optimizations by DFT calculations, the electron-cloud density of the HOMO and LUMO and their corresponding orbital energies were obtained.

II. Figures and Tables

S1. Summary of various technologies/methods for detection.

Signal generation probe/method	Signal type	Sample	Biomarkers	LOD	Range	References
Optical detection	luminescent	metabolic assay	NADPH	7.4×10^{-10} mg/ml	7.4×10^{-2} ~ 7.4×10^{-10} mg/ml	1
	colorimetric	EDTA-2Na bioassay	HBsAg	2.6×10^{-12} mg/ml	2.6×10^{-7} ~ 2.6×10^{-13} mg/ml	6
	fluorescent	Si-rhodamine	GSH	6.1×10^{-2} mg/ml	1.2 ~ 6.1×10^{-2} mg/ml	7
	inverse fluorescence trandduction	DNA sensor array based biochip	FluA	10^{2} copied/μl	~10^{5} ~ 10^{2} copied/μl	8
	SPR	Ag-coated Au nanostars	PSA	10^{-15} mg/ml	10^{-10} ~ 10^{-15} mg/ml	9
	SERRS	Au@pNIPAM hydrogel	Pyocyanin	2.1×10^{-13} mg/ml	2.1×10^{-3} ~ 2.1×10^{-13} mg/ml	10
	digital ELISA	femtoliter-volume well array	PSA	1.4×10^{-11} mg/ml	10^{-5} ~ 10^{-11} mg/ml	11
	plasmonic ELISA	Au NPs	PSA; p24	10^{-15} mg/ml	10^{-12} ~ 10^{-16} mg/ml; 10^{-12} ~ 10^{-16} mg/ml	12
Mechanical detection	hybrid mechanical nanosensor	microcantilever	CEA protein	10^{-13} mg/ml	10^{-9} ~ 10^{-14} mg/ml	15
	surface-stress sensor	PEG-coated cantilever	serum; vancomycin; oritavancin	6.6×10^{-4} mg/ml; 1.5×10^{-4} mg/ml; 1.8×10^{-7} mg/ml	66 ~ 6.6×10^{-4} mg/ml; 1.5 ~ 1.5×10^{-4} mg/ml; 1.8×10^{-2} ~ 1.8×10^{-7} mg/ml	16
	mass sensor	fluid-filled microcantilever	goat anti-mouse IgG	10^{-10} mg/ml	10^{-1} ~ 10^{-10} mg/ml	17
Electrical detection	aptamer-FET	DNA-FET	serotonin; dopamine; glucose; S1P	2.1×10^{-12} mg/ml; 1.5×10^{-12} mg/ml; 1.8×10^{-12} mg/ml; 4.0×10^{-12} mg/ml;	2.1×10^{-2} ~ 2.1×10^{-12} mg/ml; 1.5×10^{-2} ~ 1.5×10^{-12} mg/ml; 1.8×10^{-2} ~ 1.8×10^{-12} mg/ml; 4.0×10^{-2} ~ 4.0×10^{-12} mg/ml	18
	electrical characteristics	microfluidic purification chip	PSA; CA15.3	2×10^{-12} mg/ml; 15 units/ml	10^{-11} ~ 10^{-12} mg/ml; 50 ~ 15 units/ml	2
Other methods	IP-MS	plasmonic biomarkers	amyloid-β markers	5.4×10^{-13} mg/ml	1.5×10^{-12} ~ 5.4×10^{-13} mg/ml	19

Table S1. The summary of various technologies/methods for biomarkers.

Signal generation probe/method	Signal type	Signal Amplitude	Markers	LOD	Range	References
SOP-optical fibre	ASE intensity change	~$\Delta I/I$ = 80%	TNT	5×10^{-6} mg/ml	/	27
Si microtoroid	resonant wavelength shift	~ 1 pm	interleukin-2	7.5×10^{-14} mg/ml	7.5×10^{-2} ~ 7.5×10^{-14} mg/ml	28
Er:SiO_2 toroid-shaped structure	frequency splitting/beat frequency	~30 MHz; ~100 MHz	nanoparticles; viruse	single nanoparticle; single viruse	/	29
CdS-MgF_2-Ag film	nanolaser intensity change	$\Delta I/I$ = 30%	DNT	0.67×10^{-6} mg/ml	~8×10^{-6} ~ 4×10^{-6} mg/ml	30

Table S2. The summary of microcavity/resonator for detection.

SPR: Surface Plasmon Resonance; SERRS: Surface Enhanced Resonance Raman Scattering; ELISA: Enzyme Linked Immunosorbent Assay; FET: Field Effect Transistor; IP-MS: Immunoprecipitation-Mass Spectrometry; EDTA: Ethylene Diamine Tetraacetic Acid; PEG: Polyethylene Glycol; NADPH: Nicotinamide Adenine Dinucleotide Phosphate; HBsAg: Hepatitis B Surface Antigen; GSH: Glutathione; FluA: Human Influenza A Virus; PSA: Prostate Specific Antigen; p24: HIV-1 Capsid Antigen p24; CEA: Carcinoembryonic Antigen; Goat anti-mouse IgG: Goat anti-mouse Immunoglobulin-c; S1P: Sphingosine-1-Phosphate; CA15.3: Carbohydrate Antigen 15.3; SOP: Semiconducting Organic Polymers; ASE: Amplified Spontaneous Emission; TNT: 2,4,6-Trinitrotoluene; DNT: 2,4-Dinitrotoluene.

*The references of Table S1 and S2 are listed in main text. [1-2, 6-12, 15-19, 27-30]

S2. Introduction of coumarin derivatives (DC and C7).

Coumarins are a family of very large and extensively studied compounds. From the viewpoint of molecular engineering, the structure of coumarin is a two-ring system, consisting of a phenyl group fused with a pyrone ring with totally six peripheral *C-H* sites and a *C=O* and a rigid *C=C* unit. The *C=C* bond is fixed in the cis-conformation, thus helping to avoid the trans-cis transformation of normal *C=C* bonds in vinylic compounds and contributes to the strong fluorescence emission and good photostability of coumarins. Although the parent coumarin exhibits relatively weak fluorescence, lots of properly substituted coumarin derivatives yield sufficient fluorescence in visible light range.

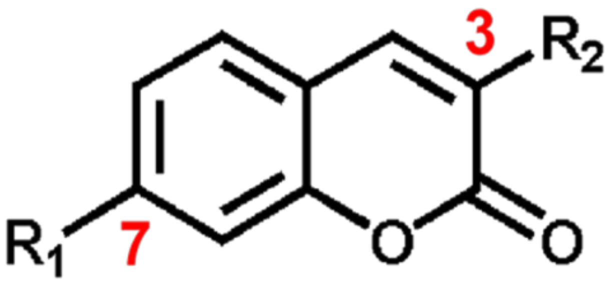

Figure S1. Molecular structure of coumarin derivatives. Electron-withdrawing groups in position 3 (R_1) or electron-donor groups in position 7 (R_2) all result in a bathochromic

emission. Next, the two molecules DC and C7 will be introduced in detail.

The synthesis process of DC can be seen as below[1]:

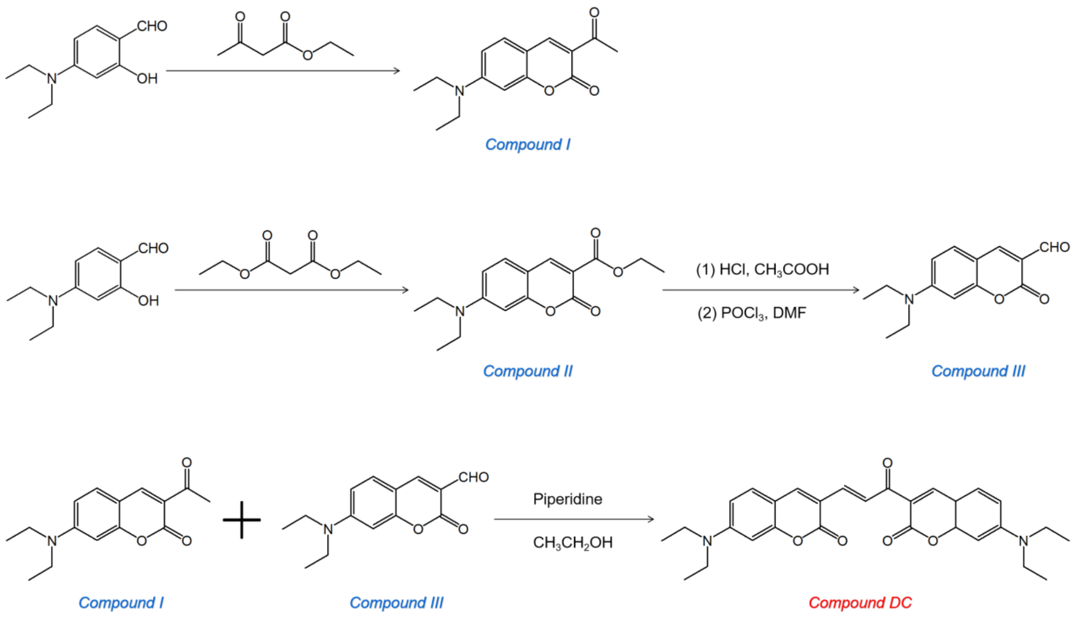

Figure S2. The synthesis process of DC[1].

Synthesis of compound I: A mixture of 4-diethylamino salicylaldehyde (1.9 g), ethyl acetoacetate (3 ml), and piperidine (1 ml) were dissolved in ethanol (50 ml) and heated to reflux for 12 h. The precipitation was collected and washed with ethanol. It was further purified by recrystallization from ethanol to afford an atrovirens solid (1.6 g).

Synthesis of compound II: A mixture of 4-diethylamino salicylaldehyde (1.9 g), diethylmalonate (3 ml), and piperidine (1 ml) were dissolved in ethanol (50 ml) and heated to reflux for 12 h. The precipitation was collected and washed with ethanol. It was further purified by recrystallization from ethanol to afford a yellow crystalline solid (1.8 g).

Synthesis of compound III: Compound II (1.45 g) in 60 ml 1:1 mixture of hydrochloric acid and acetic acid was heated to reflux for 7 h. After cooling, the pH value was adjusted to 4-5 with 45 % strength sodium hydroxide. The crystalline precipitate was filtered off, thoroughly washed with water and dried in vacuum at 50 °C. 10 ml of DMF and 2 ml of $POCl_3$ were mixed and stirred for 2 h at 50 °C under N_2 atmosphere. A suspension of obtained solid in 3 ml of DMF was then added, the mixture was warmed to 60 °C for 2 h and poured out onto ice water. The crystalline precipitate was filtered off, thoroughly washed with water and dried in vacuum at 50 ºC, which afforded *compound III* (0.46 g) as an orange solid.

Synthesis of compound DC: 3-acetyl-7-diethylaminocoumarin (2.6 g) and 7-diethylaminocoumarin-3-carbaldehyde (2.6 g) were dissolved in ethanol (100 ml), and then three drops of piperidine were added as a catalyst. The mixture was heated to reflux for 48 h, and the solvent was removed under reduced pressure. The resulting residue was then purified by chromatography on silica gel and the eluent was dichloromethane. DC was obtained as a red solid (800 mg).

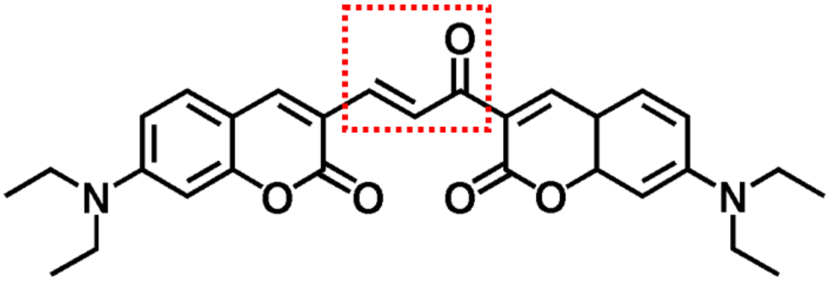

Figure S3. Molecular structure of DC compound. R_1 and R_2 of DC compounds are diethylamino *(Et_2N)* and 7-diethylaminocoumarin-3-carbaldehyde-3-acetyl group, respectively. In the 7-diethylaminocoumarin-3-carbaldehyde-3-acetyl group, the ketene bond (red dotted box) can react with homocysteine (Hcy) by Michael addition mechanism.

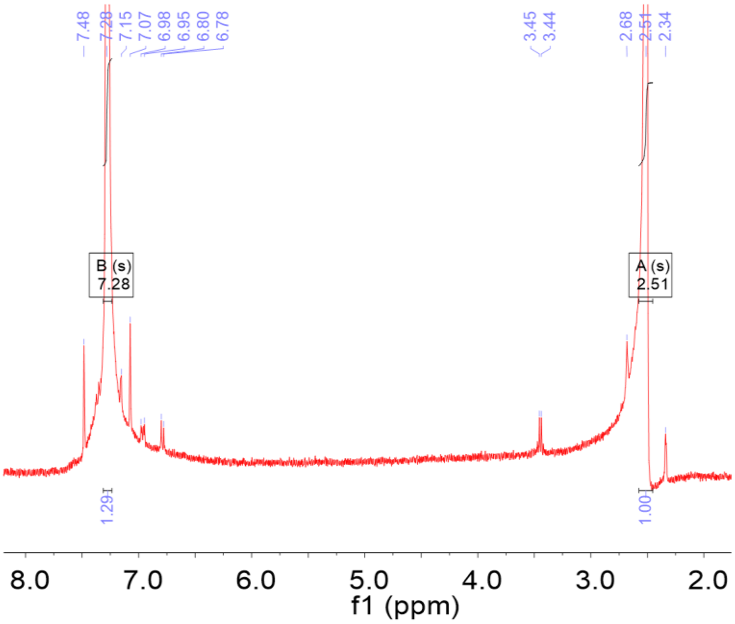

Figure S4. ^{1}H NMR spectra of DC. ^{1}H NMR (400 MHz, DMSO, ppm): 7.28 (s, 8H), 2.51 (s, 3H).

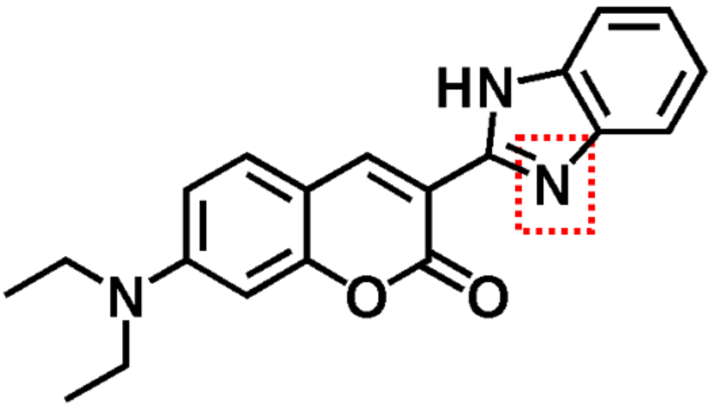

Figure S5. Molecular structure of C7 compound. R_1 and R_2 of C7 compounds are diethylamino *(Et_2N)* and 2-benzimidazolyl group, respectively. In the 2-benzimidazolyl group, the nitrogen atom (red dotted box) can supply lone-pair electrons to interact with protonic acid group.

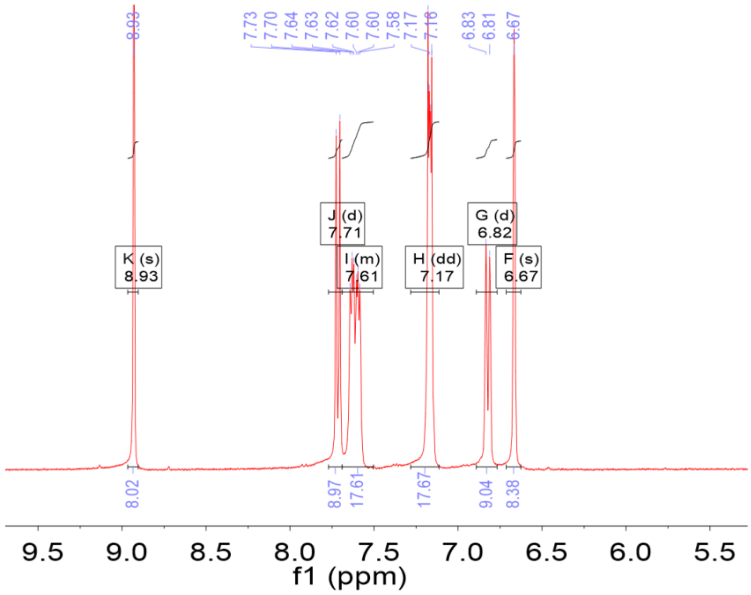

Figure S6. ^{1}H NMR spectra of C7. ^{1}H NMR (400 MHz, DMSO, ppm): 8.93 (s, 1H), 7.71 (d, 2H), 7.61 (m, 6H), 7.17 (dd, 2H), 6.82 (d, 2H), 6.67 (s, 1H).

S3. Introduction of biomarkers (Hcy and AEP).

Biological thiols, such as homocysteine (Hcy), cysteine (Cys) and glutathione (GSH) can play important effects in various physiological processed including metabolism, protein synthesis, and detoxification.

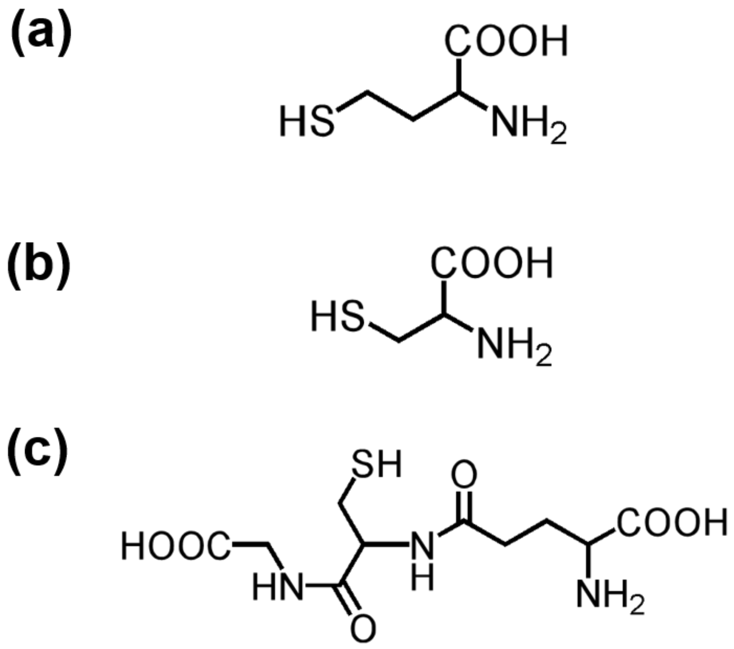

Figure S7. Molecular structure of Hcy (a), Cys (b) and GSH (c) molecule. Their inherently structures (Hcy, Cys, and GSH) are similar, and mainly be governed by the reactivity of the thiol group in Michael additions.

The selectivity of DC for Hcy was shown as below.

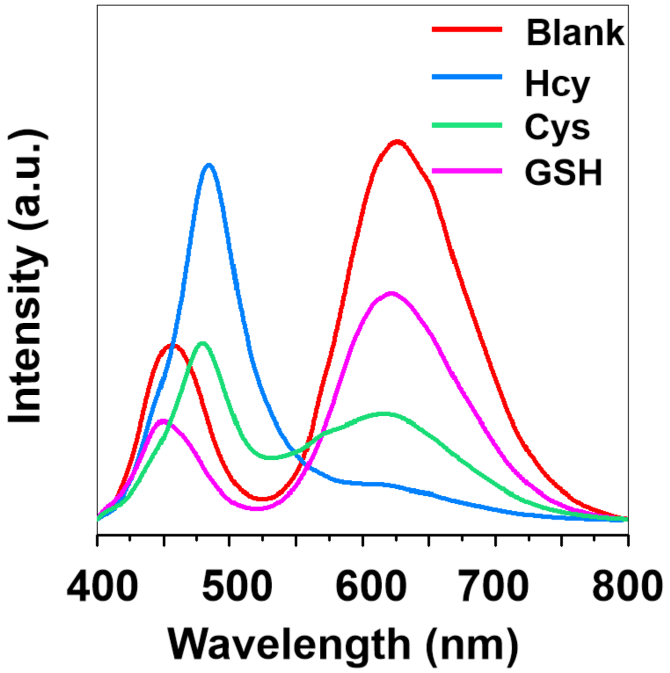

Figure S8. PL spectra of DC with different biological thiols. As shown here, the presence of Hcy induces a multi-fold fluorescence enhancement at 485 nm and the strong blue fluorescence can be observed under 375 nm UV laser. However, much slower fluorescence changes are observed with the addition of Cys, and almost no changes are observed with the addition of GSH. Thus, it is obvious that the probe DC has significant responses to Hcy and

responds relatively slow and weak to Cys and GSH. The possible mechanism of the selectivity is speculated that the different spatial interactions of the biothiols influenced the kinetics of the Michael addition. Hcy solution: The solution was prepared according to the previous report.[1] A series of concentrations of Hcy (1-10^{-9} mg/ml) was step-by-step prepared by solving Hcy powder into DMSO-PBS solution (PBS, 1×; v/v, 5/5).

Asparaginyl endopeptidase (AEP) is an endo/lysosomal cysteine endopeptidase with a preference for an asparagine or aspartate residue at the P1 site and plays an important role in the maturation of toll-like receptors 3/7/9.[2] P1 inferred C-terminal peptide bond of aspartate residue (Figure S9a).

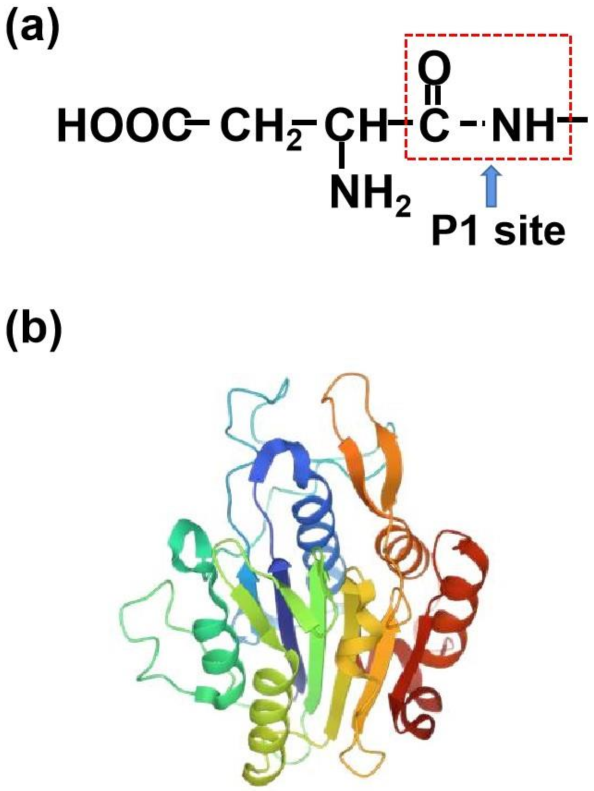

Figure S9. **a)** Structure of aspartate residue; **b)** Crystal structure of AEP/Legumain activated at pH 3.5.[2] AEP is known to undergo autoproteolytic maturation at acidic pH for catalytic activation. Here, we describe crystal structures of the mature forms of AEP. During maturation, autoproteolytic cleavage of AEP's cap domain opens up access to the active site on the core domain. Unexpectedly, an intermediate autoproteolytic maturation stage was discovered at approximately pH 3.5. This unique feature was confirmed by the crystal structure above (AEP was matured at pH 3.5). Activated AEP solution: The solution was prepared according to the previous report[2]. Autoactivation of AEP was performed by diluting aliquots the AEP proenzyme into a buffer containing 0.2 mol/L sodium citrate/citric acid and 1 mol/L dithiothreitol that was adjusted to pH 3.5. A series of concentrations of AEP (10^{-6}-10^{-17} mg/ml) was step-by-step prepared by solving AEP stock solution (Biotech. Com.) into activated solution.

S4. C7 Calculation.

The electronic structure of the protonated C7 (protonic C7 complex) was investigated using density functional theory (DFT) in Gaussian 09, where the geometry had been optimized under vacuum.

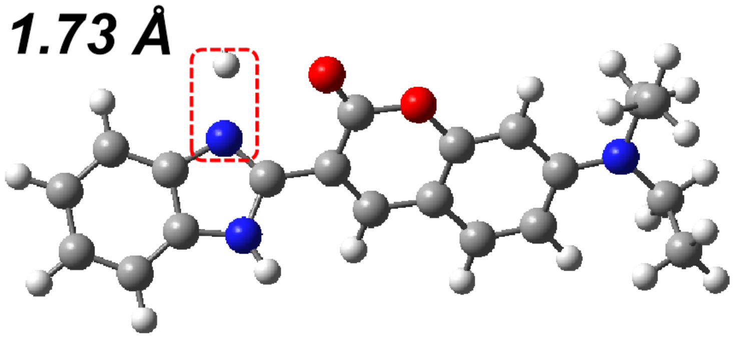

Figure S10. Optimized molecular structure of the protonic C7 complex. The optimized steric configuration of the C7-H^+ complex reveals that the distance between the N atom in C7 and the H atom is 1.73 Å, which is much shorter than the distance of a moderate hydrogen bond (~2.0 Å). These results originate from the relatively strong reaction interaction between C7 and protonic acid group, which has a direct impact on the electron-cloud density in C7 molecule.

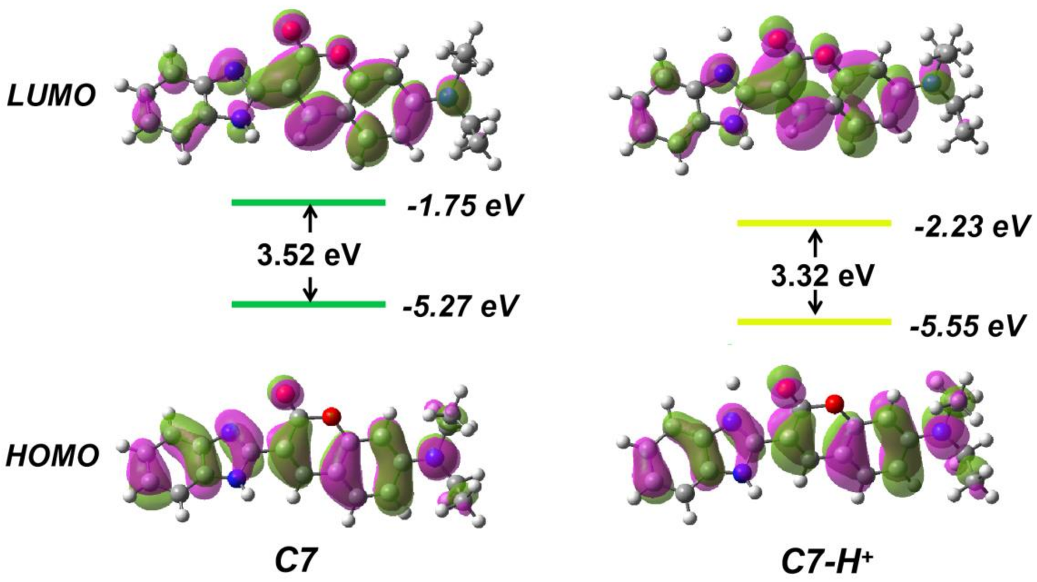

Figure S11. Pictorial presentation of the HOMOs and LUMOs in the C7 molecule and protonic C7 complex. The highest occupied molecular orbital (HOMO) density of neutral C7 is delocalized on the entire molecule, while the lowest unoccupied molecular orbital (LUMO) situates mainly on the benzimidazole and the adjacent lactone group. The H^+ binding leaves the LUMO relatively unchanged, while the HOMO's electron cloud on the benzimidazole and the adjacent lactone group decreased, indicating that the intramolecular-charge transfer (ICT) character of the protonic C7 is enhanced compared with that of the neutral C7. Consequently, the interaction between C7 and protonic acid group reduces the HOMO-LUMO gap from 3.52 to 3.32 eV, which demonstrated the theoretical feasibility of protonic acid group tuning the fluorescent emission of C7 molecules.

S5. Fabrication processes of SPP cavities.

An emulsion-solvent-evaporation method was used to fabricate dye doped organic polystyrene (PS) microcavities.[3]

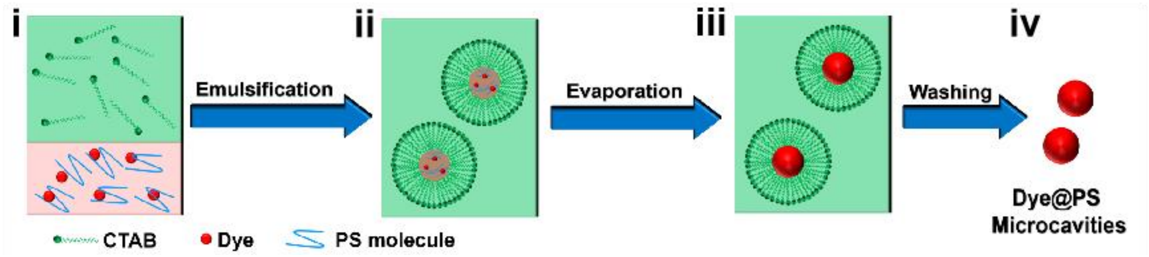

Figure S12. Schematic diagram of the fabrication processes for the dye-embedded PSS (dye@PSS) microcavities. In a typical experiment shown above, a well-mixed dye/polystyrene/CH_2Cl_2 solution was added into a cetyltrimethylammonium bromide aqueous solution (i). Then, an oil-in-water emulsion can be formed after vigorous stirring. The hydrophobic dye/PS/CH_2Cl_2 solution would be encapsulated into the hydrophobic interior of the CTAB micelles (ii). Driven by interfacial tension, PS molecules with low crystallinity prefer to aggregate into spherical structures after complete evaporation of CH_2Cl_2 solvent (iii). Finally, after washing with water several times, dye@PSS microcavities were fabricated (iv).

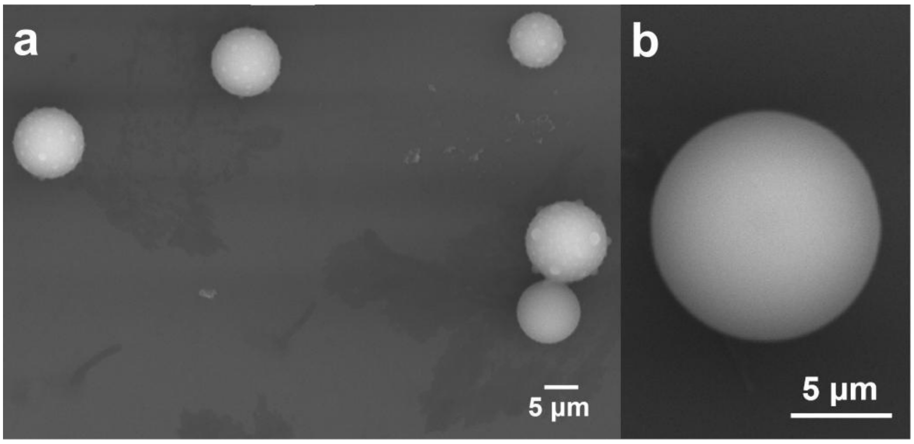

Figure S13. a) SEM image of the Dye+PSS microcavities after transferred on a substrate; Overall-view (b) of a typical microcavity. As seen here, the prepared microcavities have a perfect circular boundary and ultra-smooth surfaces, which are favorable for WGM resonance. The single perfect cavity-shaped structure can be seen more clearly by the enlarged SEM image (b).

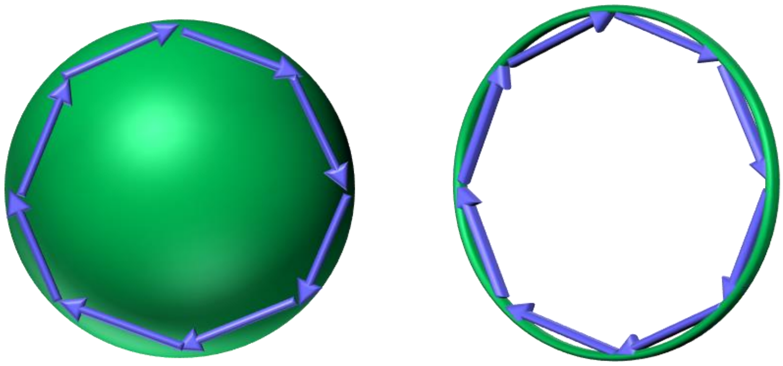

Figure S14. Overall- (left) and 2D- (right) scheme of the light transmission. The WGM optical cavity uses total reflection at the interface between the medium (about n=1.60) and the air (n=1.00) to pass photons along a circular boundary, which in turn causes traveling wave interference, thereby providing optical modulation over a wide spectral range.

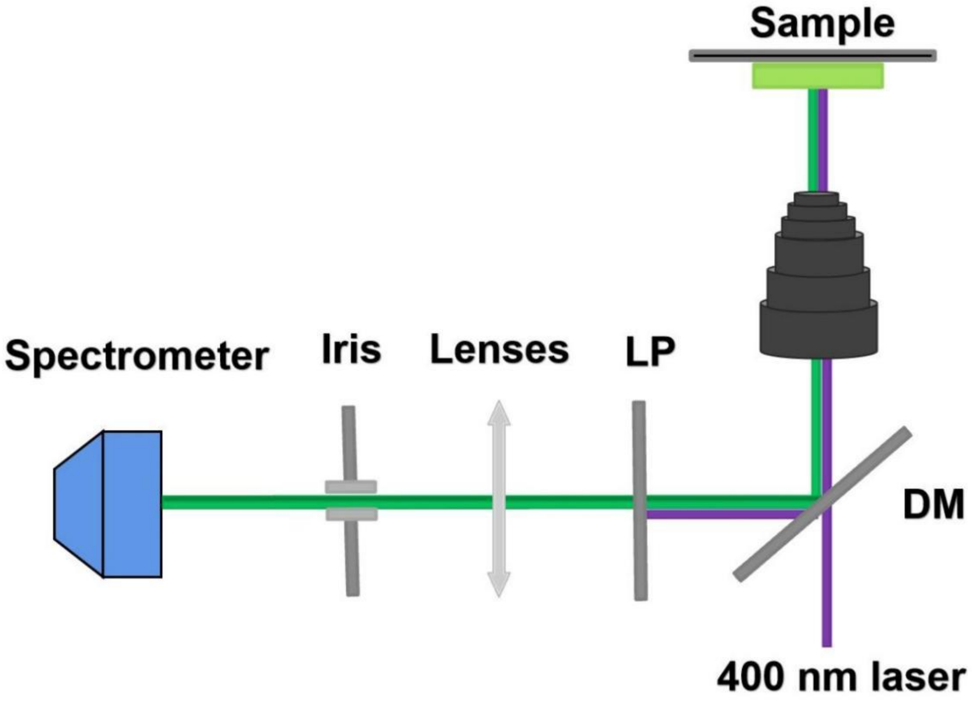

Figure S15. Schematic illustration of the experimental setup for the optical characterization. A homemade micro-photoluminescence system was used to characterize the optical performance of the PS spheres. A 400 nm pulse laser beam (35 fs, 1000 Hz) was used to pump the microlasers through an objective (Nikon CFLU Plan, ×20, N.A.=0.5). The photoluminescence signal was collected by the same microscope objective, passed through the dichroic mirror (DM, 400 nm), then a 435 nm long-pass (LP) emission filter to eliminate the exciter light, focused by a group of lenses onto a confocal iris. The output signal can be spatially selected by the iris and recorded using a spectrometer.

S6. Lasing characteristics and details of dye+PSS.

Same as in Figure 2a, a series of spectra with increasing pump power for DC+PSS can be more clearly seen in Figure S16.

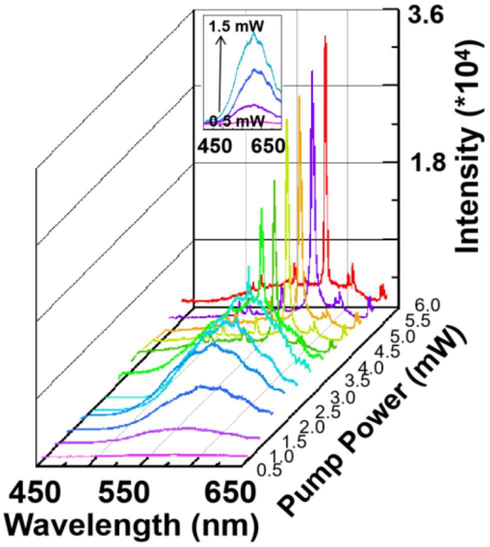

Figure S16. Lasing characteristics of spectral evolution with varied pump power for DC+PSS.

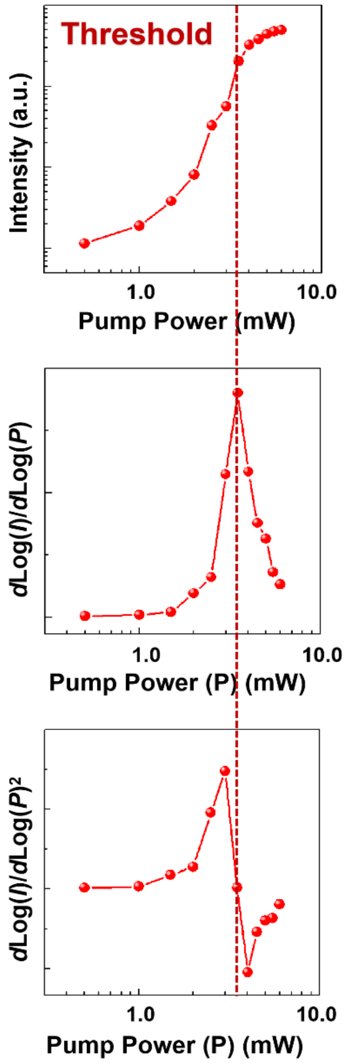

Figure S17. Log-Log plot of light-in in which the solid spheres are the experimental data (upper); The first (middle) and second (bottom) order derivative curve of DC+PSS. The red dash line indicates the threshold of the cavities. The spontaneous emission coupled to the lasing mode is as shown in Figure S17 (upper). For a small laser with a large spontaneous emission, a better way to define the threshold is to calculate the second-order derivatives of the light-in. The maximum of the first-order derivative curve shows the threshold clearly, as shown in Figure S17 (middle and bottom). Finally, the threshold of the laser is estimated to be 3.5 mW (see the red dash line in Figure S17).

Table S3. Details of splitting peak of DC+PSS.

Pump Power (mW)	6.0		5.5		5.0		4.5		4.0		3.5	
Position (nm)	590.77	592.64	590.47	592.42	590.47	592.22	590.40	592.22	590.46	592.38	590.48	592.33
FWHM (nm)	1.63	1.82	2.12	2.45	2.05	2.51	2.08	2.51	2.08	2.66	1.92	2.26
Integral Area Intensity	49446	17666	48633	28244	45135	27394	20500	27394	32511	20029	20500	10068
Pump Power (mW)	3.0		2.5		2.0		1.5		1.0		0.5	
Position (nm)	590.46	592.22	590.56	592.82	590.48	593.53	588.90	594.76	587.24	592.54	588.10	592.96
FWHM (nm)	1.43	3.56	2.51	3.16	3.29	1.40	3.20	4.52	1.69	2.67	2.88	2.53
Integral Area Intensity	5637	4079	3270	1139	810	380	382	619	108	261	110	112

Same in Figure 2d, a series of spectra evolution (including intensity) with increasing pump power for Hcy+DC+PSS can be more clearly seen in Figure S18.

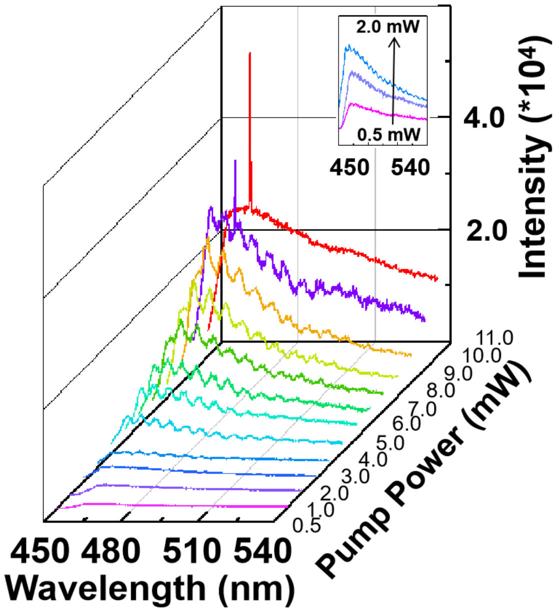

Figure S18. Lasing characteristics of spectral evolution with varied pump power for Hcy+DC+PSS.

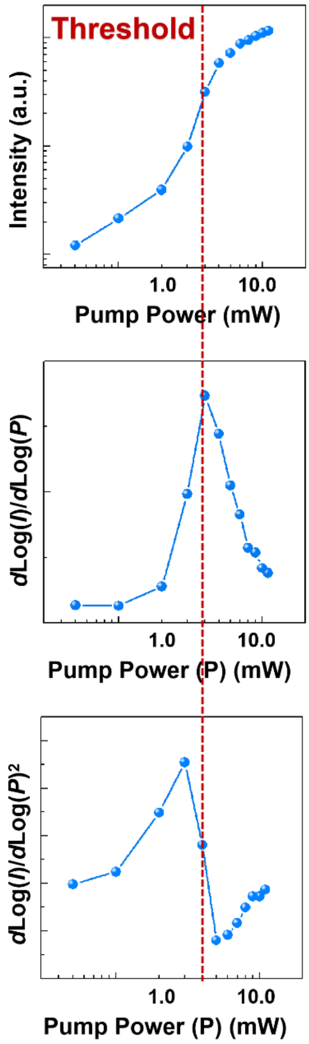

Figure S19. Log-Log plot of light-in in which the solid spheres are the experimental data (upper); The first (middle) and second (bottom) order derivative curve of Hcy+DC+PSS. The red dash line indicates the threshold of the cavities. The spontaneous emission coupled to the lasing mode is as shown in Figure S19 (upper). For a small laser with a large spontaneous emission, a better way to define the threshold is to calculate the second-order derivatives of the light-in. The maximum of the first-order derivative curve shows the

threshold clearly, as shown in Figure S19 (middle and bottom). Finally, the threshold of the laser is estimated to be 3.0 mW (see the red dash line in Figure S19).

Table S4. Details of splitting peak of Hcy+DC+PSS.

Pump Power (mW)	11.0		10.0		9.0		8.0		7.0		6.0	
Position (nm)	466.43		467.39	466.03	466.90	465.27	466.99	465.4	467.27	465.48	466.95	465.18
FWHM (nm)	0.39		2.7	0.42	1.91	1.39	2.13	1.07	2.3	1.09	2.18	0.9
Integral Area Intensity	11551		11017	4735	10382	3696	9508	2527	8784	1430	7193	1085
Pump Power (mW)	5.0		4.0		3.0		2.0		1.0		0.5	
Position (nm)	467.25	466.04	467.53	466.13	466.49	464.85	469.44	465.39	469.61	466.93	468.58	466.80
FWHM (nm)	2.71	1.33	2.05	1.39	2.08	1.56	2.05	2.1	1.6	1.85	1.05	1.35
Integral Area Intensity	5846	831	2794	865	992	364	279	358	242	298	81	121

Same in Figure 2g, a series of spectra evolution (including intensity) with increasing pump power for C7+PSS can be more clearly seen in Figure S20.

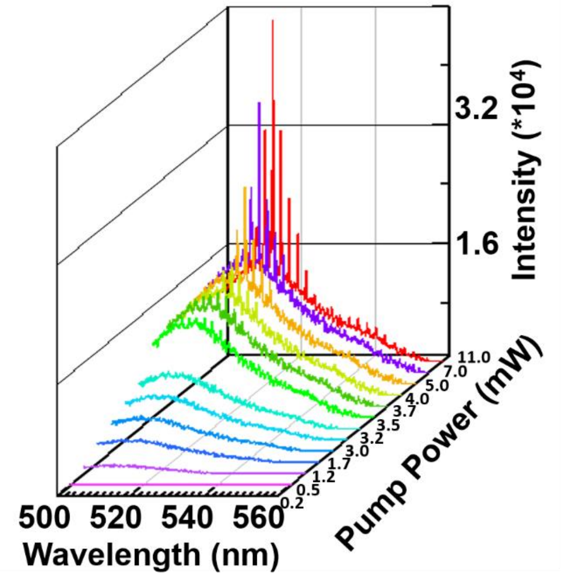

Figure S20. Lasing characteristics of spectral evolution with varied pump power for C7+PSS.

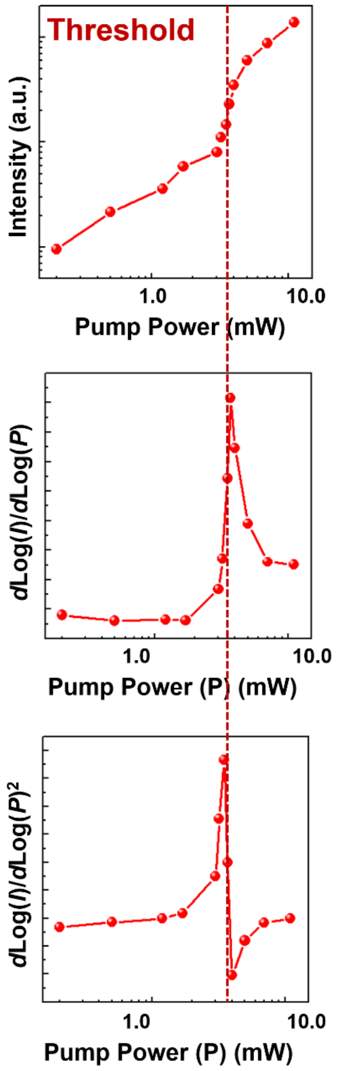

Figure S21. Log-Log plot of light-in in which the solid spheres are the experimental data (upper); The first (middle) and second (bottom) order derivative curve of C7+PSS. The red dash line indicates the threshold of the cavities. The spontaneous emission coupled to the lasing mode is as shown in Figure S21 (upper). For a small laser with a large spontaneous emission, a better way to define the threshold is to

calculate the second-order derivatives of the light-in. The maximum of the first-order derivative curve shows the threshold clearly, as shown in Figure S21 (middle and bottom). Finally, the threshold of the laser is estimated to be 3.5 mW (see the red dash line in Figure S21).

Table S5. Details of splitting peak for C7+PSS.

Pump Power (mW)	11.0		7.0		5.0		4.0		3.7		3.5	
Position (nm)	514.45	513.91	514.41	514.72	514.40	513.63	514.42	513.66	514.51	513.46	514.53	513.54
FWHM (nm)	0.32	0.27	0.23	0.55	0.32	0.65	0.38	0.64	0.73	0.33	0.62	0.39
Integral Area Intensity	12106	1661	6974	1308	4722	596	2945	536	2006	323	1158	303
Pump Power (mW)	3.2		3.0		1.7		1.2		0.5		0.2	
Position (nm)	515.65		514.62		515.07		514.96		514.54		514.28	
FWHM (nm)	1.57		2.04		1.6		1.58		0.92		1.6	
Integral Area Intensity	1102		800		582		358		215		95	

Same in Figure 2j, a series of spectra evolution (including intensity) with increasing pump power for AEP+C7+PSS can be more clearly seen in Figure S22.

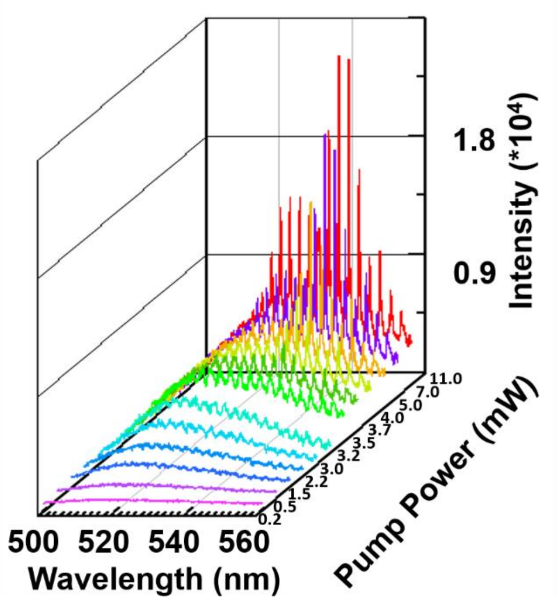

Figure S22. Lasing characteristics of spectral evolution with varied pump power for AEP+C7+PSS.

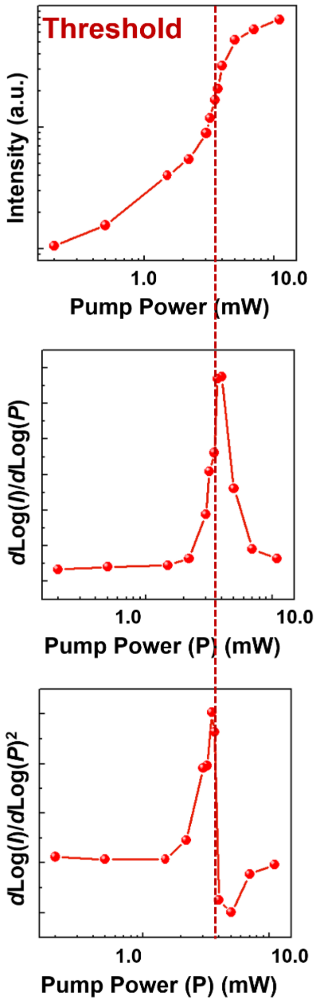

Figure S23. Log-Log plot of light-in in which the solid spheres are the experimental data (upper); The first (middle) and second (bottom) order derivative curve of AEP+C7+PSS. The red dash line indicates the threshold of the cavities. The spontaneous emission coupled to the lasing mode is as shown in Figure S23

(upper). For a small laser with a large spontaneous emission, a better way to define the threshold is to calculate the second-order derivatives of the light-in. The maximum of the first-order derivative curve shows the threshold clearly, as shown in Figure S23 (middle and bottom). Finally, the threshold of the laser is estimated to be 3.0 mW (see the red dash line in Figure S23).

Table S6. Details of splitting peak for AEP+C7+PSS.

Pump Power (mW)	11.0		7.0		5.0		4.0		3.7		3.5	
Position (nm)	540.99	541.24	540.86	541.0	540.76	540.90	540.67	541.17	540.71		540.84	
FWHM (nm)	0.21	0.63	0.27	0.96	0.26	1.11	0.33	1.11	0.92		1.21	
Integral Area Intensity	5578	2112	3837	2553	2730	2478	1811	1379	2072		1678	
Pump Power (mW)	3.2		3.0		2.2		1.5		0.5		0.2	
Position (nm)	540.27		540.16		540.04		540.06		540.13		540.28	
FWHM (nm)	1.52		1.38		1.38		1.39		1.27		1.61	
Integral Area Intensity	1183		893		547		399		156		105	

S7. Details of PL spectra with detection sensitivity.

Same in Figure 3a, a series of concentration-dependent PL spectra (including intensity) with increasing Hcy concentration for Hcy+DC can be more clearly seen in Figure S24.

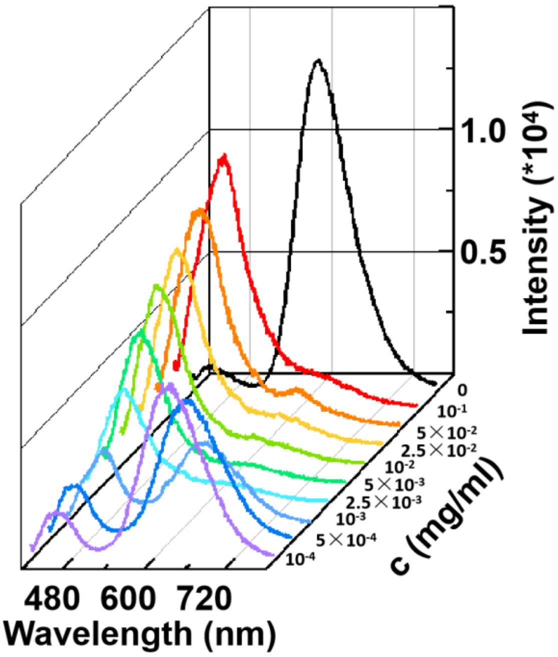

Figure S24. Hcy concentration-dependent PL spectra with varied Hcy concentration in DC. S/N = 10×log {(14000-300)/[(2000-400)-(1100-300)]} ≈ 12.

Table S7. Details of splitting peak for Hcy+DC.

Concentration (mg/ml)	10^{-1}	5.0×10^{-2}	2.5×10^{-2}	10^{-2}	5.0×10^{-3}	2.5×10^{-3}
Position (nm)	489.4	484.8	481.3	480.6	479.6	480.3
FWHM (nm)	70	67.7	64.9	65.3	64.5	61.8
Integral Area Intensity	617209	533859	449079	416082	353163	235032
10^{-3}	5.0×10^{-4}	10^{-4}	0			
472.4	459.6	461	615.1			
50.9	45.2	46	89.8			
130401	95095	80337	1268889			

Same as in Figure 3d, a series of concentration-dependent PL spectra (including intensity) with increasing Hcy concentration for Hcy+DC+PSS can be more clearly seen in Figure S25.

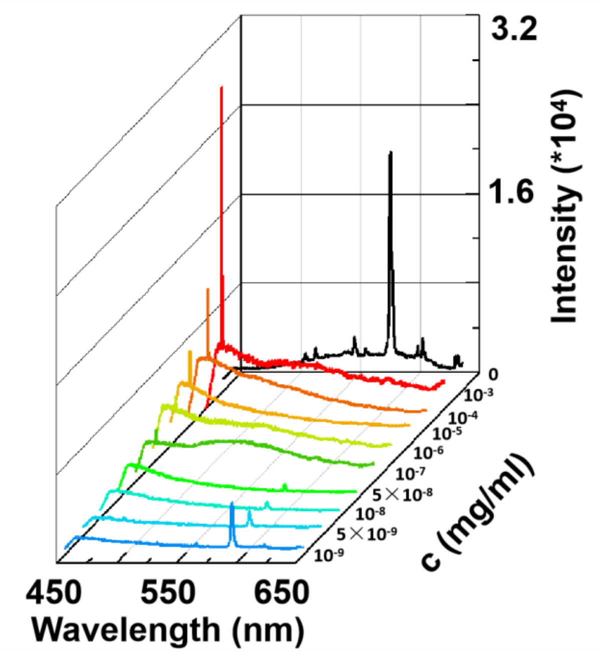

Figure S25. Hcy concentration-dependent PL spectra with varied Hcy concentration in DC+PSS. S/N = 10×log {(21200-3200)/(1600-600)} ≈ 12.

Table S8. Details of splitting peak of Hcy+DC+PSS.

Concentration (mg/ml)	10^{-3}	10^{-4}	10^{-5}	10^{-6}	10^{-7}	5×10^{-8}
Position (nm)	460~480	460~480	460~480	460~480	460~480	460~480
Integral Area Intensity	9445	7685	5092	3742	2747	2294
10^{-8}	5×10^{-9}	10^{-9}	0			
460~480	460~480	460~480	590.56			
1793	1439	904	31027			

Same as in Figure 3g, a series of concentration-dependent PL spectra (including intensity) with increasing AEP concentration for AEP+C7 can be more clearly seen in Figure S26.

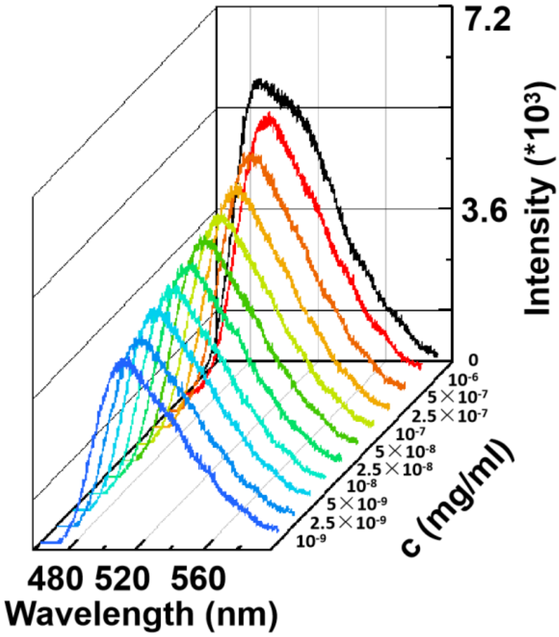

Figure S26. AEP concentration-dependent PL spectra with varied AEP concentration in C7. S/N = 10×log {(6200-1200)/(4100-1100) } ≈ 3.

Table S9. Details of splitting peak for AEP+C7.

Concentration (mg/ml)	10^{-6}		5.0×10^{-7}		2.5×10^{-7}		10^{-7}		5.0×10^{-8}		2.5×10^{-8}	
Position (nm)	534.4	504.5	533.5	504.5	533.3	504.4	533.6	504.5	534.6	504.9	533.7	504.5
FWHM (nm)	55.1	32.9	57.3	33.1	56.5	32.2	57.7	33.3	56.9	33.2	56.2	32.5
Integral Area Intensity	170708	122636	165984	110536	15735 0	101106	150061	99081	142419	99497	137400	91517

10^{-8}		5.0×10^{-9}		2.5×10^{-9}		10^{-9}		0					
533.8	504.7	533.4	504.3	534.2	504.6	534.5	504.5	520.9	491.9				
57.1	32.7	56.2	32.0	56.8	32.6	56.2	32.8	61.4	25.7				
134606	89736	131731	84676	124120	82770	119868	81764	274210	80546				

Same as in Figure 3j, a series of concentration-dependent PL spectra (including intensity) with increasing AEP concentration for AEP+C7+PSS can be more clearly seen in Figure S27.

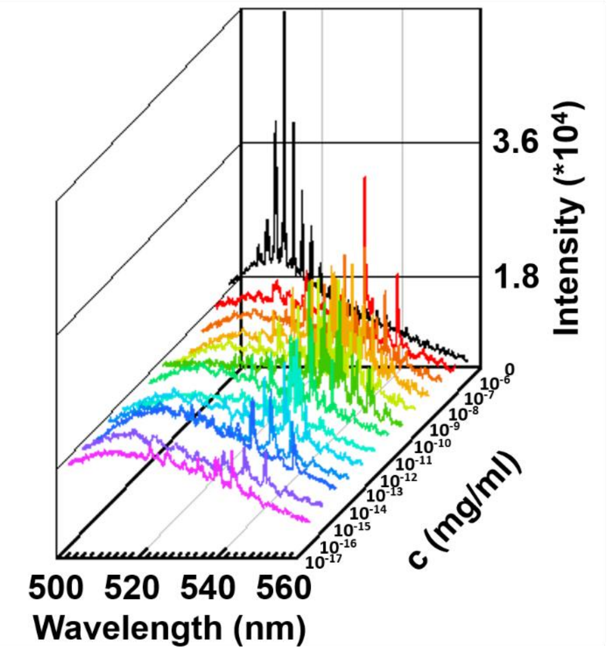

Figure S27. a) AEP concentration-dependent PL spectra with varied AEP concentration in C7+PSS. S/N = 10×log {(21200-3200)/(1600-600)} ≈ 12.

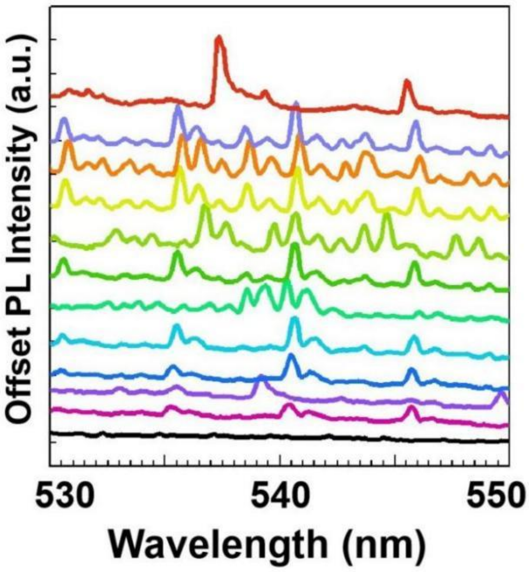

b) The zoomed-in version of Figure 3j.

Table S10. Details of splitting peak of AEP+C7+PSS.

Concentration (mg/ml)	10^{-10}				10^{-11}				10^{-12}				10^{-13}			
Position (nm)	536.93	540.90	544.92		530.67	535.87	540.87	546.14	539.58	540.53	541.41		535.67	540.85	546.12	
Integral Area Intensity	4414.8	4091.4	4639.7		1877.5	3224.9	4450.2	2393.9	3329.1	3296.5	3368.6		2239.9	3528.1	2487.5	
Concentration (mg/ml)	10^{-14}				10^{-15}				10^{-16}							
Position (nm)	535.52	540.72	546.03		539.42	549.97			535.44	540.62	1808					
Integral Area Intensity	1527.9	2929.4	1692.4		3572.5	1869.9			915.1	1873.1	546.0					
Concentration (mg/ml)	0															
Position (nm)	532.42	534.89	537.31	539.81	542.36	544.79	547.41									
Integral Area Intensity	299	209.3	263.5	271.6	236.8	205.9	122.6									

S8. Calculation of number (N) of PSSs and biomarkers:

- **Number of PSSs:**

(1) The original dye+PSS solution is prepared as described in Fig. S12, the concentration and other parameters of PSS are calculated as follows:

concentration (PS): 10.0 mg/ml; molecular weight (PS): 3.0×10^5 g/mol;

molecular weight (PS monomer): 104 g/mol; diameter of a PSS: 10^4 nm.

The molarity of PS molecules:

c_{PS} = (10.0 mg/ml) / (3.0×10^5 g/mol) = 3.3×10^{-5} mmol/ml;

The number of PS molecules:

N_{PS} = (3.3×10^{-5} mmol/ml) × (6.02×10^{23} counts/mol) × 10^{-3} mol/mmol = 2.0×10^{16} counts/ml;

The volume of a PSS:

$V_{PSS} = 4\pi r^3/3 = 4 \times 3.14 \times (5\times10^3\ nm)^3 / 3 = 5.2\times10^{11}\ nm^3 = 520\ mm^3$

The volume of a PS molecule:

V_{ps} = (3×10^5 g/mol / 104 g/mol) × (0.154 nm + 0.134 nm) × (0.15 nm + 0.5 nm) × 2 × 0.108 nm × 2 = 233 nm^3;

The number of PS molecules included in a PSS (Assuming that PSS is filled with the PS molecules):

$N_{PS\text{-}2} = V_{PSS} / V_{PS}$ = 5.2×10^{11} nm^3 / 233 nm^3 =2.2×10^9 counts/PSS;

The number of PSSs:

N_{PSS} = 2.0×10^{16} counts/ml / 2.2×10^9 counts/PSS =9.1×10^6 PSSs/ml.

(2) *The* original dye+PSS solution is then diluted for further usage. When the biomarker solution is prepared at a varied concentration, the number of PSS is accordingly:

At the concentration range from 10^{-14} to 10^{-16} mg/ml of AEP, the number of PSSs is

N_{PSS} = 9.1×10^6 PSSs/ml / 1000 = 9.1×10^3 PSSs/ml;

- **Number of activated AEP:**

concentration (AEP): 10^{-16} mg/ml; molecular weight (AEP): ~36 kDa;

At the concentration level of 10^{-16} mg/ml, The molarity of AEP molecules:

c_{AEP} = (10^{-16} mg/ml) / (3.6×10^4 mg/mmol) = 2.8×10^{-21} mmol/ml;

N_{AEP} = (2.8×10^{-21} mmol/ml) × (6.02×10^{23} counts/mol) × 10^{-3} mol/mmol ≈ 2 counts/ml.

AEP (10^{-15} mg/ml): about 20 counts/ml; AEP (10^{-14} mg/ml): about 200 counts/ml;

AEP (10^{-13} mg/ml): about 2×10^{3} counts/ml; AEP (10^{-12} mg/ml): about 2×10^{4} counts/ml;

AEP (10^{-11} mg/ml): about 2×10^{5} counts/ml; AEP (10^{-10} mg/ml): about 2×10^{6} counts/ml.

S9. Lasing spectra of biomarker AEP of different concentration.

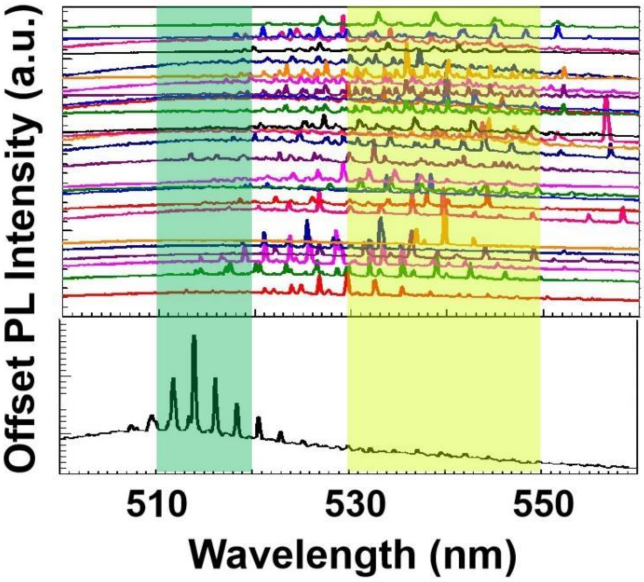

Figure S28. Lasing spectra of microcavities of C7+PSS with AEP of 10^{-6} mg/ml. The total emission intensity from cavity modes is integrated with biomarkers (green-yellow, 530-550 nm) and without biomarkers (green, 510-520 nm). The data of spectral measurements (10^{-6} mg/ml) are listed in Table S11 ($I_{10}{}^{-6}$).

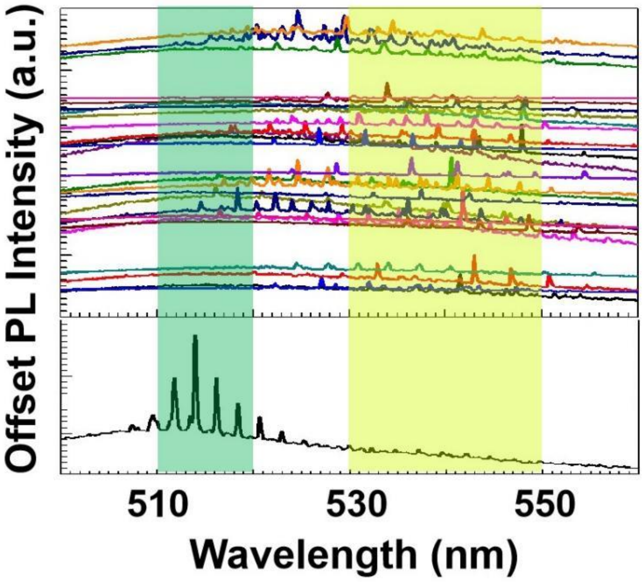

Figure S29. Lasing spectra of microcavities of C7+PSS with AEP of 10^{-8} mg/ml. The total emission intensity from cavity modes is integrated with biomarkers (green-yellow, 530-550 nm) and without biomarkers (green, 510-520 nm). The data of spectral measurements (10^{-8} mg/ml) are listed in Table S11 ($I_{10}{}^{-8}$).

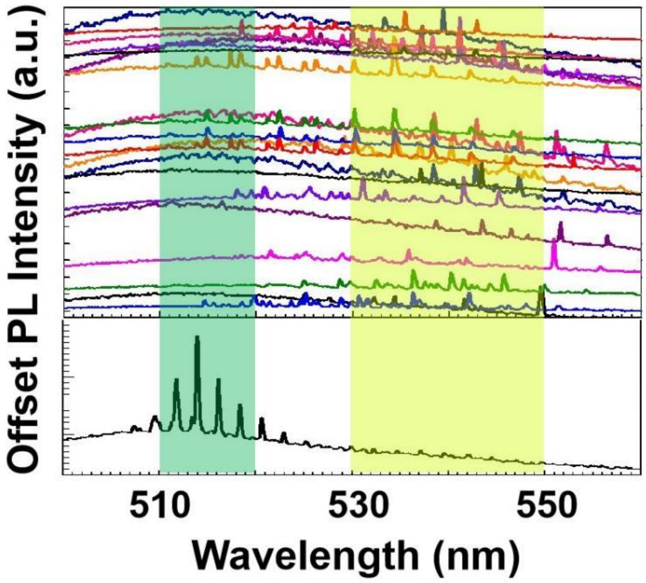

S30. Lasing spectra of different microcavities of C7+PSS with AEP of 10^{-12} mg/ml. The total emission intensity from cavity modes is integrated with biomarkers (green-yellow, 530-550 nm) and without biomarkers (green, 510-520 nm). The data of spectral measurements (10^{-12} mg/ml) are listed in Table S11 (I_{10}^{-12}).

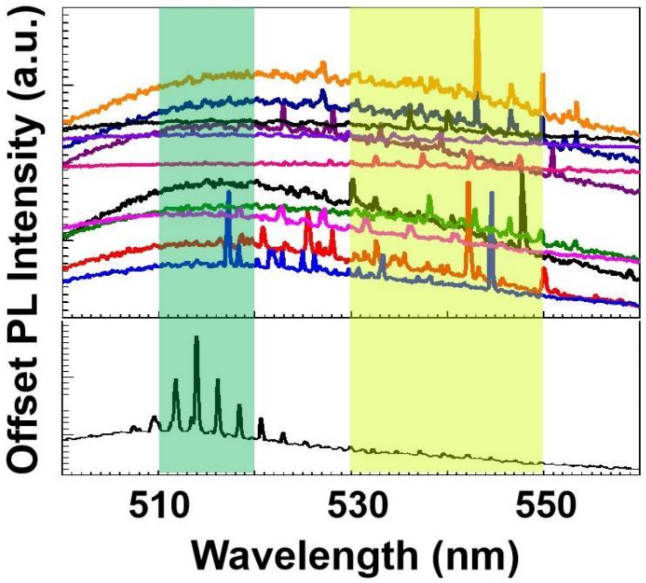

Figure S31. Lasing spectra of different microcavities of C7+PSS with AEP of 10^{-15} mg/ml. The total emission intensity from cavity modes is integrated with biomarkers (green-yellow, 530-550 nm) and without biomarkers (green, 510-520 nm). The data of spectral measurements (10^{-15} mg/ml) are listed in Table S11 (I_{10}^{-15}).

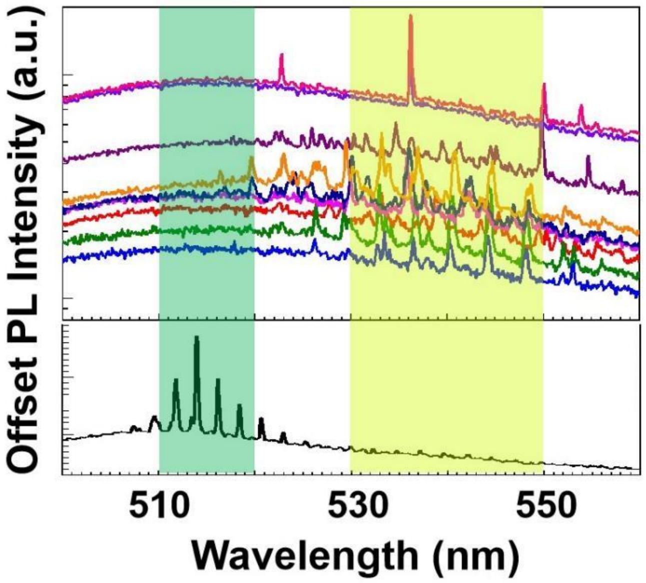

Figure S32. Lasing spectra of different microcavities of C7+PSS with AEP of 10^{-16} mg/ml. The total emission intensity from cavity modes is integrated with biomarkers (green-yellow, 530-550 nm) and without biomarkers (green, 510-520 nm). The data of spectral measurements (10^{-16} mg/ml) are listed in Table S11 (I_{10}^{-16}).

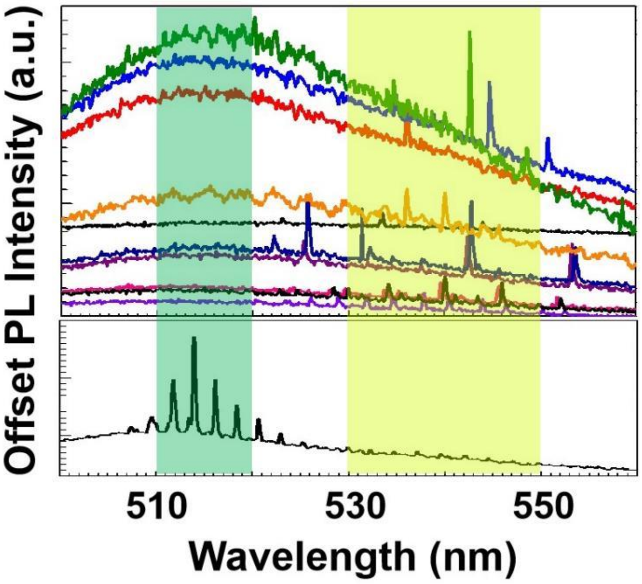

Figure S33. Lasing spectra of different microcavities of C7+PSS with AEP of 10^{-17} mg/ml. The total emission intensity from cavity modes is integrated with biomarkers (green-yellow, 530-550 nm) and without biomarkers (green, 510-520 nm). The data of spectral measurements (10^{-17} mg/ml) are listed in Table S11 ($I_{10^{-17}}$). The concentration of 10^{-17} mg/ml is the lowest density in our measurement.

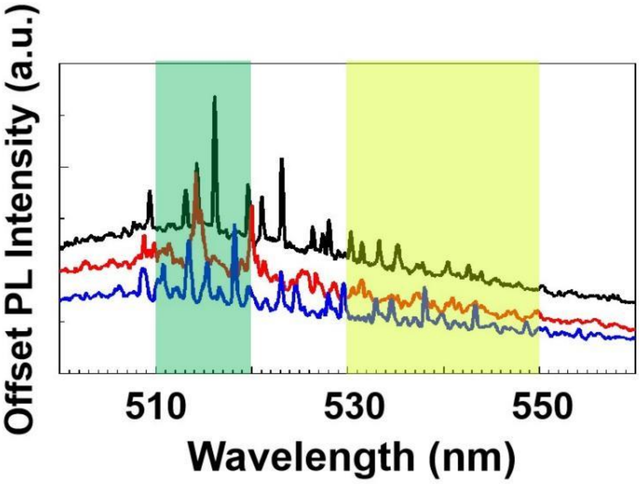

Figure S34. Lasing spectra of different microcavities of C7+PSS without AEP. The total emission intensity from cavity modes is integrated from 530 to 550 nm (green-yellow). The data of spectral measurements (0 mg/ml) are listed in Table S11 (I_0).

Table S11. The integral area intensity of spectral measurements of C7+PSS with AEP of different concentrations from 10^{-6}, 10^{-8}, 10^{-12}, 10^{-15}, 10^{-16}, 10^{-17}, 0 mg/ml.

Concentration (mg/ml)	$I_{10^{-6}}$													
Measurenment	1	2	3	4	5	6	7	8	9	10	11	12	13	14
Integral Area Intensity	13995	14451	15282	16401	18680	19865	20393	20983	21056	23752	23778	23875	24221	24989
Concentration (mg/ml)	$I_{10^{-6}}$													
Measurenment	15	16	17	18	19	20	21	22	23	24	25	26	27	28
Integral Area Intensity	25778	26708	26980	28940	29171	30698	30729	33186	33461	34399	34399	37076	38177	
														$I_{10^{-6}}$: 25287
Concentration (mg/ml)	$I_{10^{-8}}$													
Measurenment	1	2	3	4	5	6	7	8	9	10	11	12	13	14
Integral Area Intensity	3248	6280	6325	7015	7608	8544	8694	8874	9618	10360	10431	10466	10525	10752
Concentration (mg/ml)	$I_{10^{-8}}$													
Measurenment	15	16	17	18	19	20	21	22	23	24	25	26		
Integral Area Intensity	10993	11795	11838	12029	13278	13687	14622	14951	16027	16590	17301	17637		
														$I_{10^{-8}}$: 11134
Concentration (mg/ml)	$I_{10^{-12}}$													
Measurenment	1	2	3	4	5	6	7	8	9	10	11	12	13	14
Integral Area Intensity	1406	2755	2782	4006	4165	3895	5073	5582	5641	5880	5933	6151	6160	6231
Concentration (mg/ml)	$I_{10^{-12}}$													
Measurenment	15	16	17	18	19	20	21							
Integral Area Intensity	7094	7287	7492	7715	7868	7979	8908							
														$I_{10^{-12}}$: 5714
Concentration (mg/ml)	$I_{10^{-15}}$													
Measurenment	1	2	3	4	5	6	7	8	9	10	11			
Integral Area Intensity	1904	2457	2904	3119	3459	3460	3728	3894	4113	4689	5327			
														$I_{10^{-15}}$: 3550
Concentration (mg/ml)	$I_{10^{-16}}$													
Measurenment	1	2	3	4	5	6	7	8	9					
Integral Area Intensity	1074	1228	2090	2583	2851	2949	3412	4029	4678					
														$I_{10^{-16}}$: 2766

Concentration (mg/ml)	$I_{10^{-17}}$													
Measurenment	1	2	3	4	5	6	7	8	9	10				
Integral Area Intensity	342	454	999	1118	1208	1218	1254	1904	2153	2233				
														$I_{10^{-17}}$: 1288
Concentration (mg/ml)	I_0													
Measurenment	1	2	3											
Integral Area Intensity	1327	1339	1418											
														I_0 : 1362

Table S12. Intensity range I/Proportion I, Intensity range II/Proportion II and Intensity range III/Proportion III at different concentration levels of AEP.

Concentration (mg/ml)	Intensity range I ($\times10^4$)	Proportion I	Intensity range II ($\times10^4$)	Proportion II	Intensity range III ($\times10^4$)	Proportion III
10^{-6}	2.3-2.7	33%	1.9-3.1	63%	1.5-3.5	85%
10^{-8}	1.0-1.25	35%	0.75-1.5	69%	0.5-1.75	92%
10^{-12}	0.5-0.625	38%	0.375-0.75	67%	0.25-0.875	90%
10^{-15}	0.34-0.39	36%	0.29-0.44	64%	0.24-0.49	82%
10^{-16}	0.24-0.3	33%	0.18-0.36	56%	0.12-0.42	78%

From the table, it can be found that in the intensity range I, the proportion is about ~30% and the intensity distributions are not overlapping; in the intensity range II, the proportion is about ~60% and the intensity distributions are a little overlap in the low concentrations; in the intensity range III, the proportion is about ~90% and the intensity distributions are large overlap in the low concentrations.

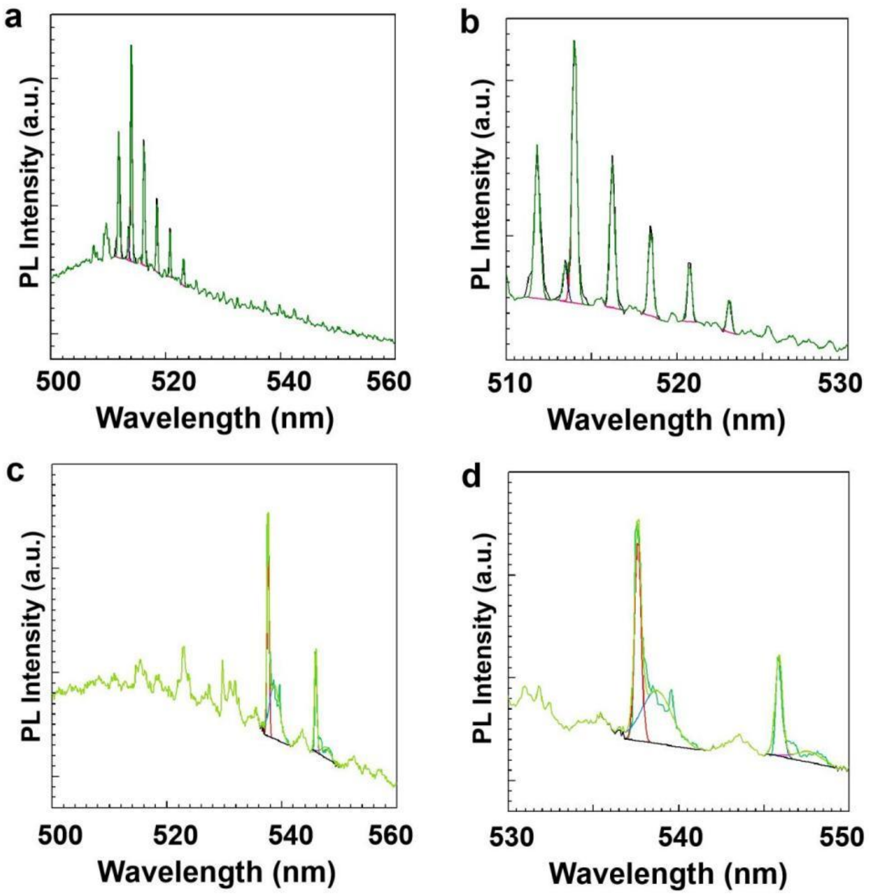

Figure S35. a) Spectral features without biomarkers (C7+PSS only) in 510-530 nm. **b)** the zoomed-in version of (a). **c)** Spectral features after reacting with biomarker AEP (AEP+C7+PSS) in the 530-550 nm. **d)** the zoomed-in version of (c).

Table S13. Details without biomarkers (C7+PSS only) in 510-530 nm.and with biomarkers (AEP+C7+PSS) in the 530-550 nm.

Biomarkers	without biomarkers						with biomarkers			
Position (nm)	511.81	513.98	516.20	518.45	520.71	523.05	537.60	538.71	545.87	547.66
FWHM (nm)	0.37	0.32	0.32	0.32	0.32	0.31	0.44	2.04	0.50	1.80
Integral Area Intensity	3961	6040	3236	1888	1292	735	9553	11529	5363	2007

S10. LOD Calculation.

As shown in Figure 4f, the enhancement constants (K_{EM}) were quantified through the Stern-Volmer equation[4]

$$(I-I_0)/I_0 = K_{EM}\times c$$

where c is the concentration of biomarkers. The slope (K_{EM}) was obtained from the linear fit of AEP concentration-dependent laser intensity (monitored in the wavelength range: 530-550 nm) curve in the low-concentration region in Figure 4f inset. I is the integrated intensity of laser peaks between 530~550 nm after the addition of the biomarkers, I_0 is the integrated intensity of laser peaks between 530~550 nm before the addition of biomarker (see Table S11). The K_{EM} for AEP is calculated to be as large as 6.4×10^{16}.

To accurately express the LOD of biomarkers, the LOD is defined as[4]

$$LOD = 3\sigma/K_{EM}$$

$$\text{Where } \sigma = 100\times(I_{SE}/I_0)$$

where σ is the standard deviation. I_{SE} is the standard deviation of the laser intensity measurement, as determined by the measurement of blank samples between 530~550 nm. On the basis of these data, the LOD was calculated to be as low as 1.4×10^{-16} mg/ml.

We measure a series of blank samples (Supporting Information Figure S34), then the integral area intensity of every measurement is as follows.

I_{SE} ={ [1327-1362)^2 + (1339-1362)^2 + (1418-1362)^2]/3}^1/2 = 40.4

$\sigma = 100\times(I_{SE}/I_0) = 100\times40.4/1362 = 3.0$

$LOD = 3\sigma/K_{EM} = 3\times3.0/(6.4\times10^{16}) = 1.4\times10^{-16}$ mg/ml.

References:

[1] K. P. Wang, S. N. Xu, Y. Lei, W. J. Zheng, Q. Zhang, S. J. Chen, H. Y. Hu, Z. Q. Hu, *Talanta* **2019**, *196*, 243.

[2] L. X. Zhao, T. Hua, C. Crowley, H. Ru, X. M. Ni, N. Shaw, L. Y. Jiao, W. Ding, L. Qu, L. W. Hung, W. Huang, L. Liu, K. Q. Ye, S. Y. Ouyang, G. H. Cheng, Z. J. Liu, *Cell Res.* **2014**, *24*, 344.

[3] C. Wei, M. M. Gao, F. Q. Hu, J. N. Yao, Y. S. Zhao, *Adv. Optical Mater.* **2016**, *4*, 1009.

[4] W. Liu, et al. *ACS Appl. Mater. Interfaces* **2017**, *9*, 16448.
